# Transcriptional drug repositioning and cheminformatics approach for differentiation therapy of leukaemia cells

**DOI:** 10.1038/s41598-021-91629-x

**Published:** 2021-06-15

**Authors:** Yasaman KalantarMotamedi, Fatemeh Ejeian, Faezeh Sabouhi, Leila Bahmani, Alireza Shoaraye Nejati, Aditya Mukund Bhagwat, Ali Mohammad Ahadi, Azita Parvaneh Tafreshi, Mohammad Hossein Nasr-Esfahani, Andreas Bender

**Affiliations:** 1 Centre for Molecular Informatics, Department of Chemistry, University of Cambridge, Lensfield Road, Cambridge, CB2 1EW UK; 2 Department of Animal Biotechnology, Cell Science Research Center, Royan Institute for Biotechnology, ACECR, Isfahan, Iran; 3 Department of Genetics, Faculty of Science, Shahrekord University, Shahrekord, Iran; 4 Molecular Medicine Department, Institute of Medical Biotechnology, National Institute of Genetic Engineering and Biotechnology, Tehran, Iran; 5 Open Analytics, Jupiterstraat 20, 2600 Antwerp, Belgium

**Keywords:** Bioanalytical chemistry, Targeted therapies, Cheminformatics, Cancer genomics, Cancer stem cells, Acute myeloid leukaemia

## Abstract

Differentiation therapy is attracting increasing interest in cancer as it can be more specific than conventional chemotherapy approaches, and it has offered new treatment options for some cancer types, such as treating acute promyelocytic leukaemia (APL) by retinoic acid. However, there is a pressing need to identify additional molecules which act in this way, both in leukaemia and other cancer types. In this work, we hence developed a novel transcriptional drug repositioning approach, based on both bioinformatics and cheminformatics components, that enables selecting such compounds in a more informed manner. We have validated the approach for leukaemia cells, and retrospectively retinoic acid was successfully identified using our method. Prospectively, the anti-parasitic compound fenbendazole was tested in leukaemia cells, and we were able to show that it can induce the differentiation of leukaemia cells to granulocytes in low concentrations of 0.1 μM and within as short a time period as 3 days. This work hence provides a systematic and validated approach for identifying small molecules for differentiation therapy in cancer.

## Introduction

Differentiation therapy has several advantages compared to chemotherapy, such as its irreversible effect and the rapid clearance of tumour bulk, following terminal maturation of blast cells^1^. One prominent example of this type of therapy is the treatment of acute promyelocytic leukaemia (APL, an aggressive type of acute myeloid leukaemia or AML) by a combination of all-trans retinoic acid (ATRA) and arsenic^1^. In acute myeloid leukaemia (AML) cells, differentiation is blocked in the cellular maturation stage^2^ which prevents leukaemia cells from terminal differentiation. It is assumed that many neoplastic cells have reversible defects in their differentiation patterns, where small molecules can cause tumour reprogramming and thereby induce terminal differentiation and apoptosis^3^. Despite the importance of differentiation therapy, selecting particular small molecules to induce such differentiation is challenging. However, the recent availability of large-scale biological data can uncover mechanisms in the differentiation process one would like to modulate to achieve this aim, with one such approach being compound selection and drug repurposing based on gene expression (transcriptomics) data.

Transcriptional drug repositioning has recently gained significant attention, both due to increased data availability, as well as several success stories that have been reported^4,5^. The key idea is that a modulation of a biological system by a process (such as a disease) should be *counteracted* by compound treatment, in the particular readout space that is available, such as transcriptomics space. This is rather distinct from single-target approaches in drug discovery, where first a target of interest is isolated and then a ligand is desired—in the case here, rather modulation of the *whole* system is taken into account. This in particular enables identifying compounds that would modulate multiple genes and pathways of a disease simultaneously. One of the key elements of transcriptional repurposing is that it is an unbiased approach and enables scientists to come up with a *testable hypothesis*, both in terms of compounds and modes of action of the compounds selected, in a disease or biological process of interest. Its application in oncology, neurodegenerative, infectious^6^ and rare diseases has enabled the identification of new indications for approved drugs^4^.

Various scoring systems exists that enable the scoring (and ranking) of compounds in the database against a disease of interest quantitatively, given a set of transcriptomics data^7^. In the absence of a gold standard to compare all scoring systems, we can only evaluate methods fully by experimental validations for the disease of interest, or, in the absence of this, by retrospective literature validation. Moreover, gene expression data is noisy and for each compound we usually have several instances of application of the compound available, with dose, time and cell line being major experimental factors to consider^8^. Some studies integrate all different instances of the same compound into a unified signature representing the compound^9^; however, given that e.g. dose/exposure is a significant factor that influences treatment response this is not the option preferred by the authors of the current work.

To address some of the challenges that still exist in the transcription layer of the data, we have integrated the transcriptional drug repositioning approach with cheminformatics approaches for incorporating protein level inference data and selecting compounds in a more informed manner. The cheminformatics side of the work benefits from learning from large scale databases of compound target pairs (385,126 pairs) and predicting activity profile of compounds in the connectivity map (CMap)^10^ database against 1,643 protein targets^11^ which is further annotated with importance of the predicted target for the disease of interest and leads to a cheminformatics based scoring system that complements the transcriptional based bioinformatics scores.

Even though connectivity map approaches have been studied before for cancer. Its application in differentiation therapy is an emerging and attractive area^12,13^. In this work, we evaluate and validate our transcriptional drug repositioning approach in the emerging application area of differentiation therapy for acute myeloid leukaemia. For this purpose, the disease state is defined as comparison of HL60 leukaemia cells compared to healthy granulocytes. This comparison raises to a blueprint of differentiation and we hypothesise that finding a compound that can target those genes effectively would induce differentiation of leukaemia cells to granulocytes. This rationale was chosen because such differentiation is known to induce apoptosis in the cancer cells, and it is hence more suitable for therapeutic purposes than more broad cytotoxic modes-of-action^14^.

**Figure 1:**
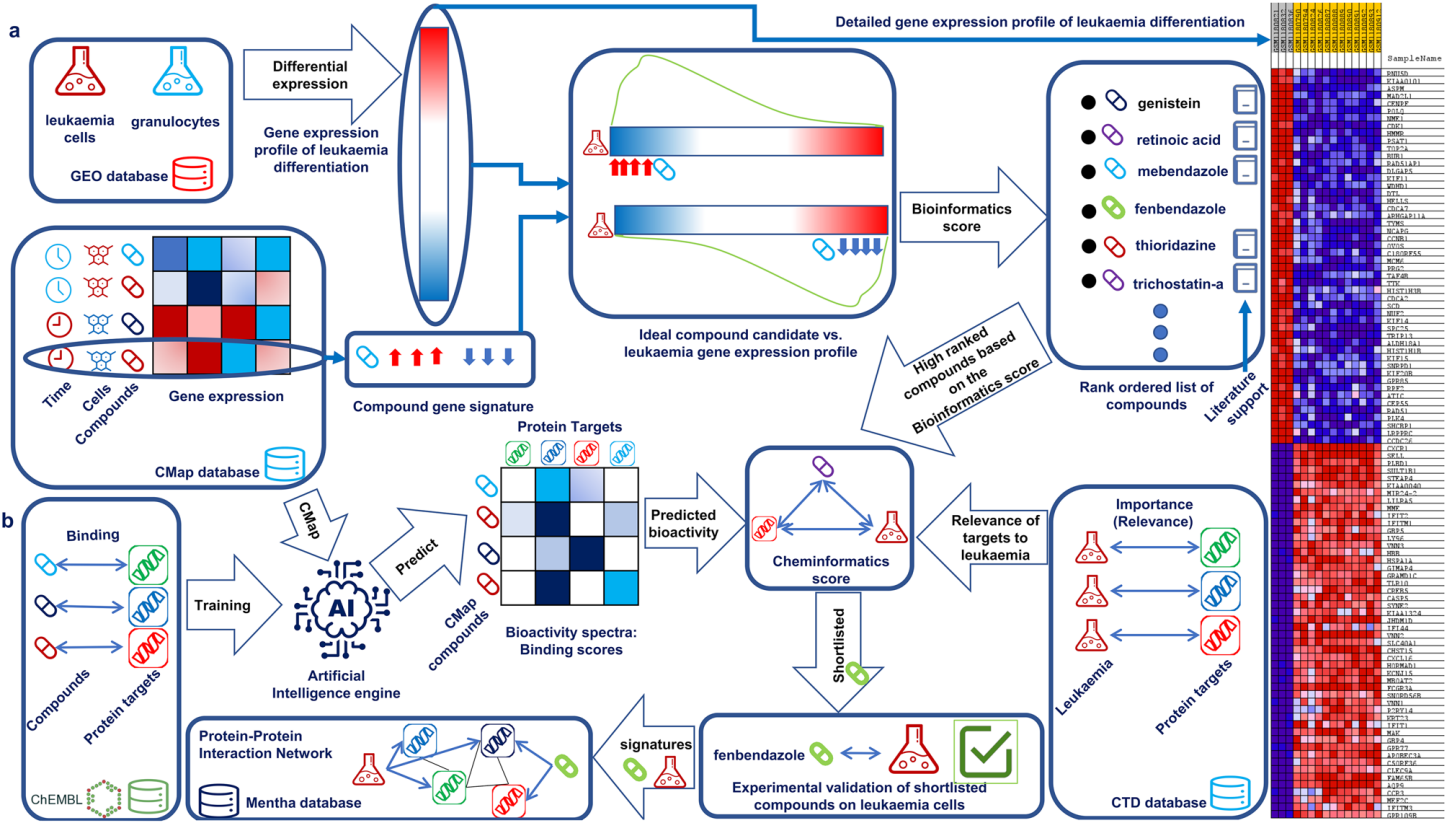
Combination of gene expression and in silico target prediction approaches for compound selection and mode-of-action analysis: (**a**) bioinformatics part of the work involves extracting disease signature from GSEO database and rank ordering differentially expressed genes as well as extracting gene signatures of compounds from the connectivity map database. Gene set enrichment analysis was used to rank order al the compounds in CMap database that can optimally reverse most dysregulated genes in the leukaemia signature. Highly ranked compounds based on the bioinformatics were supported by literature. It also displays differentially expressed genes in the leukaemia signature (on the right side). (**b**) Cheminformatics part of the work involves training an Artificial Intelligence engine on a database of compound target pairs extracted from ChEMBL and predicting binding scores for all compounds in CMap vs. a large range of protein targets. It also involves extracting inference scores that identifies relevance of each target to leukaemia. The Cheminformatics score calculates average relevance of top seven predicted targets of CMap compounds to leukaemia. Combination of the bioinformatics score and the cheminformatics score led to the identification of fenbendazole which was followed up with in-vitro validation. Gene signature and protein targets of fenbendazole as well as gene signature of leukaemia was mapped to a protein–protein interaction network to rationalise mode of action.

Our computational approach presented here, integrates transcriptional drug repositioning and cheminformatics approaches, and enables identifying compounds that can induce differentiation of leukaemia cells to granulocytes (Fig. 1). In this work we prospectively validated fenbendazole that was identified by the approach and validated that it can truly induce the differentiation of leukaemia cells to granulocytes.

## Results

Our integrated drug repositioning and cheminformatics approach was applied for selecting compounds that can induce differentiation of leukaemia cells to granulocytes. Figure 1 depicts the workflow and data sources used for this purpose. The Bioinformatics part of the work (Fig. 1a) extracts disease signature (HL60 leukaemia cells vs. granulocyte) from GEO^15^ dataset and rank orders genes based on differential expression. 5764 gene signatures extracted from connectivity map database were used to query the disease profile using Gene Set Enrichment analysis (GSEA)^16^ and rank ordered to target most differentially expressed genes of the disease profile most efficiently in a reverse way. The Cheminformatics part of the work (Fig. 1b) used an artificial intelligence (AI) engine that is based on Laplacian modified naïve bayes approach developed earlier^11^. The underlying principal is that structurally similar compounds tend to bind to similar targets and is capable of predicting targets for any compound given its structure. The AI engine trained a model on compound target pairs extracted from ChEMBL^11^ database and predicts probability of binding of all the compounds in the connectivity map database to 1643 protein targets. This facilitates incorporating all known and potential targets of each compound into the model. The model also takes into account relevance of each predicted target to leukaemia by incorporating relevance scores extracted from Comparative Toxicogenomics database (CTD)^17^. The Cheminformatics score highly ranks compounds that target most relevant protein targets of the disease estimated by averaging relevance score to leukaemia for top seven predicted targets for each compound. Moreover, protein–protein interaction network is incorporated to elucidate mode of action of selected compounds based on the bioinformatics and cheminformatics scoring system.

### Retrospective validation in leukaemia

It was found that 20 out of the 30 highest-ranked compounds from CMap with negative connectivity were supported by literature according to their relevance to leukaemia (Table 1). Notably current standard differentiation therapy for leukaemia, tretinoin (retinoic acid, ATRA) was ranked 15 based on the bioinformatics score among 5765 CMap signatures. It is also showing over the average (12.5) cheminformatics score (14.5). One example of active compounds on leukaemia cells is Thioridazine (ranked 11) which is known to inhibit proliferation and induce apoptosis in leukaemia cells, but does not affect normal lymphocytes^18^. We predicted from the cheminformatics side of the analysis that Thioridazine targets the Histamine H1 receptor, the Dopamine D1-3 receptors, and the Muscarinic acetylcholine receptors M4 and M5. Binding to all of these targets are supported in ChEMBL^19^ for *Homo sapiens* (with IC_50_ values of 0.07 µM, 0.19 µM, 0.03 µM, 0.01 µM, 0.09 µM and 0.009 µM, respectively). Interestingly, all these proteins are frequently targeted among the 50 highest-ranking compounds selected for this disease, based on gene expression data (P values: 0.1, 0.02, 0.08, 0.03, 0.03, and 0.06). Wortmannin (ranked 14) inhibits K562 myelogenous leukaemia cells proliferation and induces apoptosis by regulating survival signalling pathways, such as PI3K/Akt/Nκ-KB. This pathway is known to be important in the development of leukaemia^20^. The most probable predicted target for Wortmannin is the PI3-kinase p110-alpha subunit (PIK3CA). PIK3CA is a member of the PI3K-Akt signalling pathway, and the activity against which is reported in ChEMBL with an IC_50_ of 0.013 µM. The CMap instance of this compound (ID 6202) downregulates *TRIB3*, *FYN* and *MLST8* in the “PI3K-Akt Signalling” pathway. Among the top 30 ranked compounds based on the Bioinformatics score, 14 had over the average (12.5) cheminformatics score (Table 1). 13 out of that 14 had literature support for leukaemia and only fenbendazole, one type of benzimidazole, was novel and hence was selected for experimental validation in this work.

**Table 1:** Top 30 ranked compounds for leukaemia: the table shows the highest ranked compounds along with their predicted targets and the scores for the predicted targets (Target score).

Rank	Bioinformatics score	Cheminformatics score	Compound name	Retrospective validation for leukaemia	Retrospective validation for other cancers	Selected for experimental validation	Target 1 name	Target 1 score	Target 1 disease score	Target 2 name	Target 2 score	Target 2 disease score	Target 3 name	Target 3 score	Target 3 disease score	Target 4 name	Target 4 score	Target 4 disease score	Target 5 name	Target 5 score	Target 5 disease score	Target 6 name	Target 6 score	Target 6 disease score	Target 7 name	Target 7 score	Target 7 disease score
1	− 0.81	23	Podophyllotoxin	*^76^			EDNRB	26	30	EDNRA	21	11	F3	20	40	PDE11A	13	3	FKBP1A	13	12	CYP3A4	10	44	NR3C1	9	22
2	− 0.79	15	Leflunomide	*^77^			MTTP	21	2	APOB	19	20	DHODH	16	0	ATF1	14	2	TRPV1	13	12	NFKB1	13	46	RAF1	12	22
3	− 0.79	16	Colchicine	*^78^			TUBB1	37	3	F3	8	40	STS	5	24	BDKRB1	2	11	ALK	2	2						
4	− 0.79	5	Terazosin			*	EHMT2	20	18	CCR4	18	3	ADRA1B	18	4	ADRA1A	15	3	EHMT1	14	0	UBE2N	14	0	PDPK1	8	9
5	− 0.78	7	Prenylamine		*^79^		ADRB2	18	9	CASR	16	2	ADRB3	13	3	ADRB1	12	6	C3AR1	9	7	CAPN2	8	17	SSTR2	8	2
6	− 0.78	21	Trimethylcolchicinic acid	*^80^			TUBB1	23	3	STS	14	24	DRD1	8	0	F3	7	40	ALK	6	2	ABCC1	6	57	ACHE	5	18
7	− 0.78	29	Etoposide	*^81^			NCOA3	30	100	F3	23	40	NCOA1	20	16	RORC	15	0	TOP1	13	17	SLC5A1	12	0			
8	− 0.78	14	Mebendazole	*^21^			TEK	30	14	RAF1	20	22	KDR	17	25	F9	10	0	GRB7	6	14	CHEK2	6	22	ITK	5	3
9	− 0.78	5	Adenosine phosphate	*^82^			RSEL	99	3	IMPDH1	91	4	P2RY2	87	12	P2RY1	70	0	P2RX1	69	0	AHCY	68	12			
10	− 0.78	5	Cefoperazone				SLC22A8	72	9	SLC22A6	66	7	CMA1	44	3	ELANE	35	0	PGF	15	5						
11	− 0.77	2	Thioridazine	*^18^			HRH1	34	0	DRD1	24	0	DRD2	23	3	CHRM5	20	6	DRD3	18	0	CHRM4	18	3	HRH2	18	0
12	− 0.77	14	Nocodazole	*^83^			TEK	36	14	KDR	21	25	STK33	17	3	ITK	15	3	F9	13	0	RAF1	12	22	ABL1	9	32
13	− 0.77	4	Tetryzoline				ADRA2A	27	4	ADRA2B	24	7	NISCH	23	0	ADRA2C	23	3	BDKRB1	21	11	DRD1	12	0	ADRA1A	12	3
14	− 0.77	20	Wortmannin	*^20^			PIK3CA	72	11	MTOR	58	23	MYLK	18	17	ABCB1	10	63	SOAT2	10	3	CYP19A1	9	23	ADCY1	9	4
15	− 0.77	15	Tretinoin	*^84^			RARG	65	11	RARB	65	28	RXRA	64	12	RARA	59	20	RXRG	53	11	RXRB	53	8	RBP4	49	11
16	− 0.77	46	Genistein	*^85^			ALDH2	40	15	ESR2	25	16	ESR1	23	100	TOP2A	20	100	XDH	15	17	CYP1A1	15	38	CYP1B1	15	36
17	− 0.76	15	Ly-294002	*^86^			PRKDC	68	18	PIK3CA	38	11	PIK3CB	32	6	PIK3CD	26	24	PIK3CG	25	18	PIK3C2B	14	3	MTOR	11	23
18	− 0.76	4	Proxymetacaine			*	UBE2N	11	0	HTR4	10	0	P2RY12	6	0	PDGFRA	1	4	BCHE	1	18	CHRM4	1	3	MBTPS1	1	3
19	− 0.76	9	Sulfapyridine	*^87^			NTRK1	7	5	CYP2C18	6	10	GRM4	6	0	EDNRA	6	11	HTR6	4	0	EDNRB	4	30	PIK3C3	3	7
20	− 0.75	22	Fenbendazole		*^31^	*	TEK	27	14	AURKB	11	21	RAF1	11	22	KDR	10	25	AURKA	8	13	ITK	7	3	MCL1	5	56
21	− 0.75	4	Mephentermine				CASR	12	2	GHSR	10	4	ADRB2	8	9	ADRB1	6	6	ADRB3	5	3	TACR3	2	3	CCKBR	2	3
22	− 0.75	5	Dobutamine				ADRB2	32	9	ADRB1	31	6	ADRB3	30	3	PGF	5	5	OPRD1	5	0	OPRM1	4	4	KISS1R	4	11
23	− 0.75	11	Clenbuterol				ADRB2	27	9	ADRB1	27	6	ADRB3	18	3	BACE1	3	8	CTSD	1	25	IGF1R	0	16	CYP2D6	-1	11
24	− 0.75	2	Thioridazine (rep)	*^18^			HRH1	34	0	DRD1	24	0	DRD2	23	3	CHRM5	20	6	DRD3	18	0	CHRM4	18	3	HRH2	18	0
25	− 0.75	15	Ly-294002 (rep)	*^86^			PRKDC	68	18	PIK3CA	38	11	PIK3CB	32	6	PIK3CD	26	24	PIK3CG	25	18	PIK3C2B	14	3	MTOR	11	23
26	− 0.75	8	Trichostatin a	*^88^			HDAC6	28	3	HDAC10	25	3	HDAC1	24	17	HDAC8	23	3	HDAC2	23	9	HDAC9	23	11	HDAC11	22	7
27	− 0.74	2	Remoxipride	*^89^			DRD2	28	3	DRD3	10	0	UTS2R	9	0	HCRTR1	9	7	HCRTR2	9	0	HTR4	7	0			
28	− 0.74	5	Nadide				IMPDH1	105	4	P2RY2	90	12	RSEL	86	3	GAPDH	77	18	P2RX1	74	0	P2RY1	70	0	P2RY4	66	0
29	− 0.74	4	Terfedine	*^26^			TACR2	28	3	NPY2R	22	7	HRH1	18	0	CHRM3	11	2	KCNH2	11	10	NPC1L1	10	0	CCR5	10	5
30	− 0.74	27	Ouabain	*y^26^			KLF5	39	0	NR3C1	37	22	ATP12A	30	0	SHBG	28	7	STAT3	24	42	FGF2	23	16	FGF1	23	100

High target scores show high probability of the compound to bind to that protein target based on our *in*-silico prediction approach. The “Target disease score” shows how much the target is related to the disease according to Comparative Toxicogenomic Database (CTD). Compounds that have retrospective validation and literature support for leukaemia or other cancer types and the ones selected for experimental validation in this work are marked with a * for each relevant column. The Bioinformatics score is the score calculated based on anti-correlation of the compound gene signature and leukaemia differentiation signature. The Cheminformatics score is the rounded average of Target Disease Score for the top seven predicted targets of each compound.

A recent study explored application of transcriptional drug repositioning in differentiation therapy of leukaemia blast cells^21^. Notably, mebendazole, another member of benzimidazole family, was highly ranked in the results of that study as well as ours. It was also prospectively validated in that study that mebendazole treatment of leukaemia cells at doses of 1 μM for 9 days induced morphology changes. There are several differences in terms of computational approach and the findings. The signature in the previous study was retrieved from multiple data sets, including normal haematopoiesis, the classical model of ATRA differentiation therapy, and drugs known to modulate differentiation, and all contributed to the scoring using a computational approach called Lineage Maturation Index. However, the computational approach in this study does not include ATRA in the dataset and comes directly from the comparison of HL60 cells to granulocytes, which makes the rediscovery of mebendazole and ATRA as a current therapy more remarkable. In terms of comparison of the findings, we discover mebendazole as well in our top results along with another member of the same family, fenbendazole. However, the cheminformatics approach enabled us to select fenbendazole over mebendazole due to its more relevant predicted bioactivity profile as discussed in results. To compare the experimental side of the studies of this work and the previous work^21^, we should mention that phenotypically, mebendazole in the previous work induced morphological changes but did not induce granulocytes differentiation; however with fenbendazole in this work we show in the next section that granulocytes were formed. Fenbendazole induces the differentiation in lower doses of 0.1–0.5 μM for shorter duration of 3 days and after 7 days at dose of 0.1 μM apoptotic cells appeared. For mebendazole, full morphological changes appeared after 9 days at dose of 1 μM. However, it is difficult to compare the two studies quantitatively due to differences in the use of NBT assay (calculating absorbance in this study vs. the percentage of dark blue cells in the previous study). Etoposide is also another drug predicted in both studies which was shown to induce the differentiation with 44% of cells identified as positive in the NBT assay. Also another recent study confirmed the use of mebendazole for leukaemia patients in pre-clinical and clinical settings^22^. Another recent study used network pharmacology and applied it in the area of differentiation therapy for leukaemia^13^. Remarkably, among the compounds that came up in top results of our and their study, ATRA (our rank = 15), mebendazole (rank = 8), colchicine (rank = 3), podophyllotoxin (rank = 1), nocodazole (rank = 12) and dinoprost (rank = 230) were shared. They further confirmed that mebendazole, podophyllotoxin and dinoprost can also induce the differentiation of leukaemia cells. Moreover, a quantitative proteomics study also identifies one of our highly ranked compound genistein (rank = 16) to induce apoptosis in both MV4-11 and HL-60 cells via caspase activation^23^. Genistein is also known to induce granulocytic differentiation and DNA Strand Breakage in HL60 and K562 cells^24,25^. Terfenadine (rank = 29) and ouabain (rank = 30) in our top results were also discovered using a connectivity map approach previously for AML leukaemia and validated experimentally but were not tested for differentiation therapy^26^.

### Prospective validation on leukaemia

**Figure 2:**
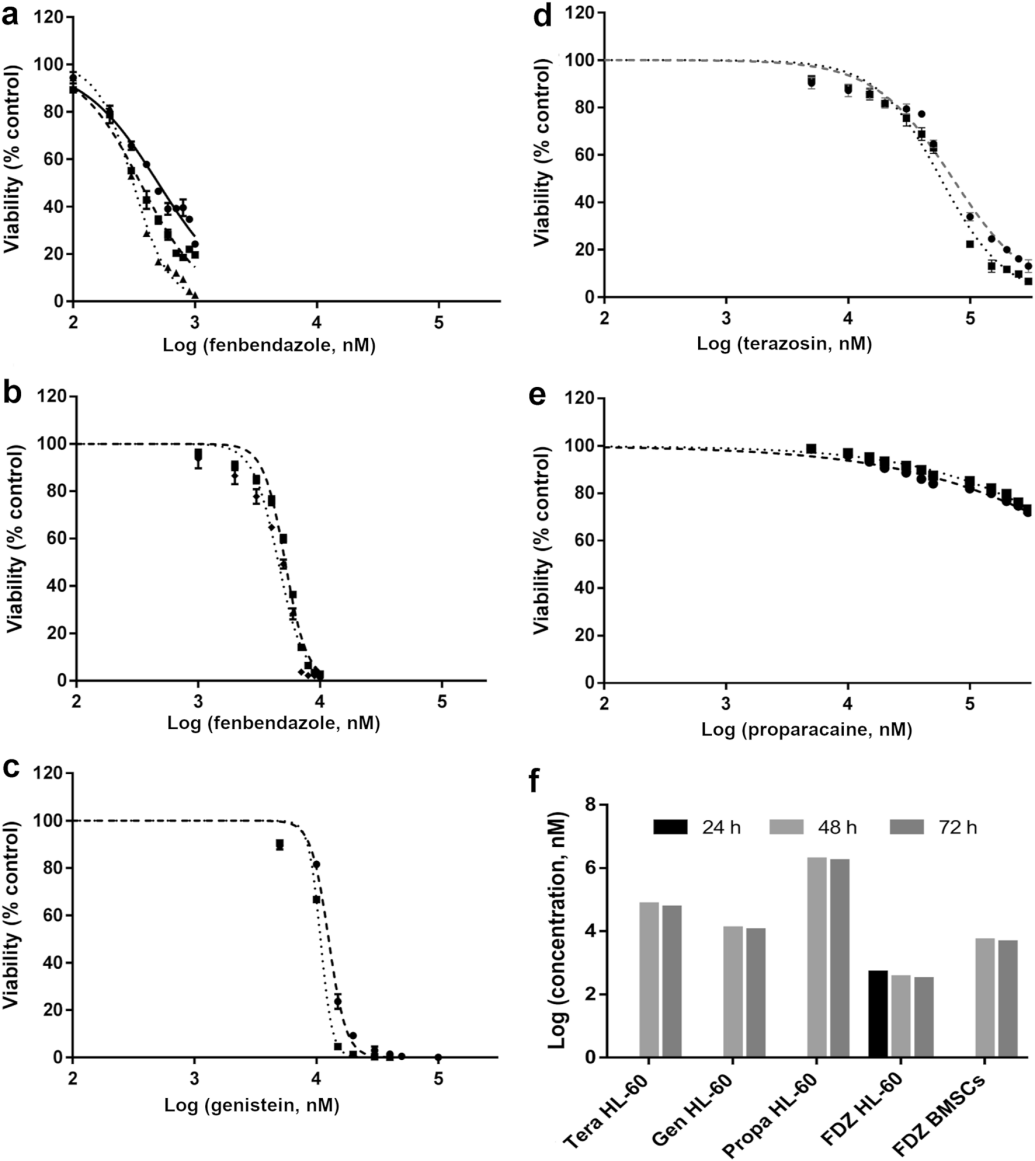
Prospective validation of selected compounds on HL60 cell line. Among the compounds tested on the HL60 cell line fenbendazole (**a**) shows highest efficacy with LC_50_ values of 0.5 µM, 0.36 µM and 0.31 µM after 24, 48 and 72 h, respectively. The LC50 of Fenbendazole on BMSC cells was 5 µM (**b**). Genistein (**c**) was only minimally active (with LC50s of 12 µM after 48 h) while terazosin (**d**) and proxymetacaine (**e**) were inactive in HL60 cells. Fenbendazole exhibited 14.5-fold selectivity at 72 h over BMSC cells (**f**).

Among the highest-ranked predictions based on the bioinformatics score without any current literature support proxymetacaine (ranked 18), fenbendazole (ranked 20) and terazosin (ranked 4) were selected to be tested in vitro on the HL60 leukaemia cell line with genistein (ranked 16) serving as a control. Here, proxymetacaine and terazosin were selected based on high bioinformatics score only, whilst fenbendazole was selected based on the combined bioinformatics- and cheminformatics-approach as it had both high bioinformatics and cheminformatics score (see Table 1). As shown in Table 1, fenbendazole is the only compound with both high bioinformatics (in the top 30) and cheminformatics score with no previous literature on leukaemia. As shown in Fig. 2a–f the prospective validation revealed that genistein (Fig. 2c) was only minimally active (with LC50s of 12 µM after 48 h) while terazosin (Fig. 2d) and proxymetacaine (proparacaine, Fig. 2e) were inactive in HL60 cells. On the other hand, fenbendazole exhibited a profound effect on HL60 cells with LC_50_ values of 0.50 µM, 0.36 µM and 0.31 µM at 24, 48 and 72 h, respectively (Fig. 2a). In order to establish functional selectivity over healthy cells, the toxicity of fenbendazole was further evaluated on human bone marrow stem cells (BMSC) and exhibited LC_50_ values of 5.1 µM and 4.5 µM after 48 and 72 h, respectively (Fig. 2b). Hence, at time point of 72 h, fenbendazole exhibits 14.5-fold selectivity in killing HL60 cells over BMSC cells was observed (Fig. 2f).

We further investigated if selectivity is driven by the slower cell cycle of BMSC cells, compared to HL60 cells. Therefore, toxicity of fenbendazole was also measured on HFF cells, which gave rise to the same LC_50_ values to the BMSCs and hence same 14.5-fold selectivity. Hence, overall, the data show that the LC_50_ values of fenbendazole were ca. 35 times lower than that of the positive control, genistein (with LC_50_ values of 12.5 µM and 10.8 µM at 48 and 72 h, respectively). Moreover, fenbendazole exhibits 14.5-fold selectivity of cancer cells over somatic cells.

**Figure 3:**
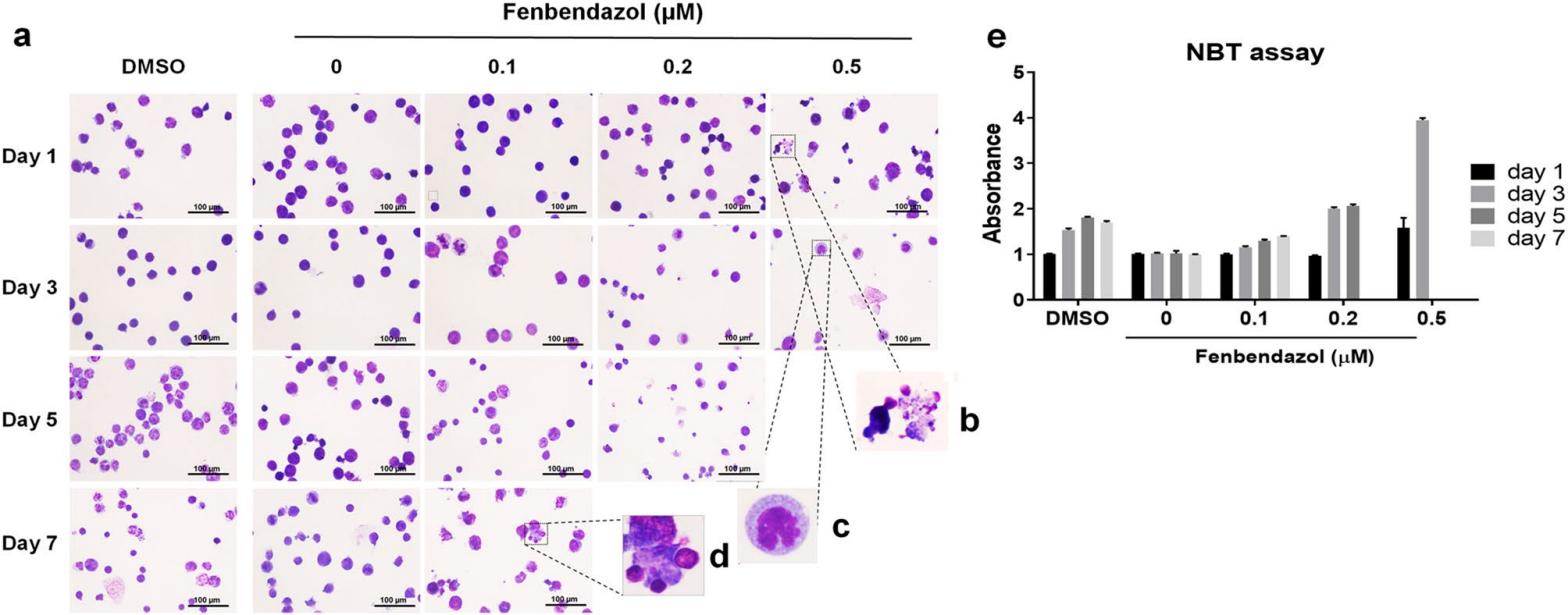
Fenbendazole-induced differentiation of HL60 cells to granulocytes followed by cell death: (**a**) Cell morphology at 1, 3, 5 and 7 days post treatment with 0.1 µM, 0.2 µM and 0.5 µM of fenbendazole via staining with Wright–Giemsa; (**b**) necrotic cells; (**c**) granulocytes; (**d**) apoptotic cells. (**e**) Different concentrations of fenbendazole were employed in the NBT reduction assay at different time points. The assay demonstrates that 0.5 µM of fenbendazole increased NBT reduction upon neutrophil differentiation significantly compared to positive control treatment with DMSO). Hence it can be concluded that the mode-of-action hypothesis fenbendazole inducing cell death via differentiation to granulocytes is supported by experiment.

Next, the mode-of-action of fenbendazole on HL60 cells was investigated by cellular imaging in 1, 3, 5 and 7 days after treatment with three different concentrations (0.1, 0.2 and 0.5 µM) of fenbendazole via staining with Wright–Giemsa (Fig. 3a) in order to determine the possible transformation of leukaemia cells to the granulocyte lineage as predicted. After 1 day in the presence of 0.5 µM fenbendazole, we found a heterogeneous cell population of apoptotic cells (marked by nuclear fragmentation and apoptotic body formation, Fig. 3d) along with necrotic cells (marked by intact nuclei and increased cell volume, Fig. 3b) and cells with a lobulated nucleus. The latter indicates the presence of granulocytes (Fig. 3c). In the presence of 0.2 µM of fenbendazole, many indented cells were observed after 1 day of treatment. After day three, the 0.1 µM-treated cells revealed nuclear indentation while lobulated cells were seen at 0.2 µM concentration. On the other hand, most of the cells incubated with 0.5 µM fenbendazole underwent cell death while others showed multi-lobed nucleus. Apoptotic morphology was observed in 0.2 µM and 0.1 µM-treated cells after 5 and 7 days, respectively (Fig. 3d). Hence, it can be concluded that at lower concentrations, the majority of cells appear to go through apoptosis via induced differentiation to granulocytes which is consistent with our prediction. However, in higher concentrations of fenbendazole treatment (around LC50) we found a mixture of three physiological events (apoptosis, necrosis and neutrophil differentiation) after a short time (day 1). It seems that in this concentration, some of the cells are killed directly, which may occur by necrosis or apoptosis, and unspecific effects are frequently observed at such higher concentrations. Comparison of these findings with 1.25% DMSO treatment, as a well-known inducer of granulocytic differentiation^27^, revealed a similar response at a later time point in case of DMSO treatment (on day 7), compared to 0.1 and 0.2 µM fenbendazole-treatment at an earlier time point (day 5, Fig. 3a). An NBT reduction assay, as a marker of granulocyte and monocyte differentiation^24^, was used to quantitatively verify the hypothesis of granulocytic differentiation. It was revealed that an acute treatment with fenbendazole (0.5 µM) resulted in a sharp increment in NBT reduction upon neutrophil differentiation (Fig. 3e). We observed a moderate (but significant) increase in absorbance of formazan deposits by lower concentrations of fenbendazole during the time. These findings were comparable to positive control treatment with DMSO, which hence supported the hypothesis of a transformation of leukaemia cells to granulocytes (Fig. 3e). Taken together, the results obtained are consistent with the hypothesis that fenbendazole induces cell death via induction of differentiation.

**Figure 4:**
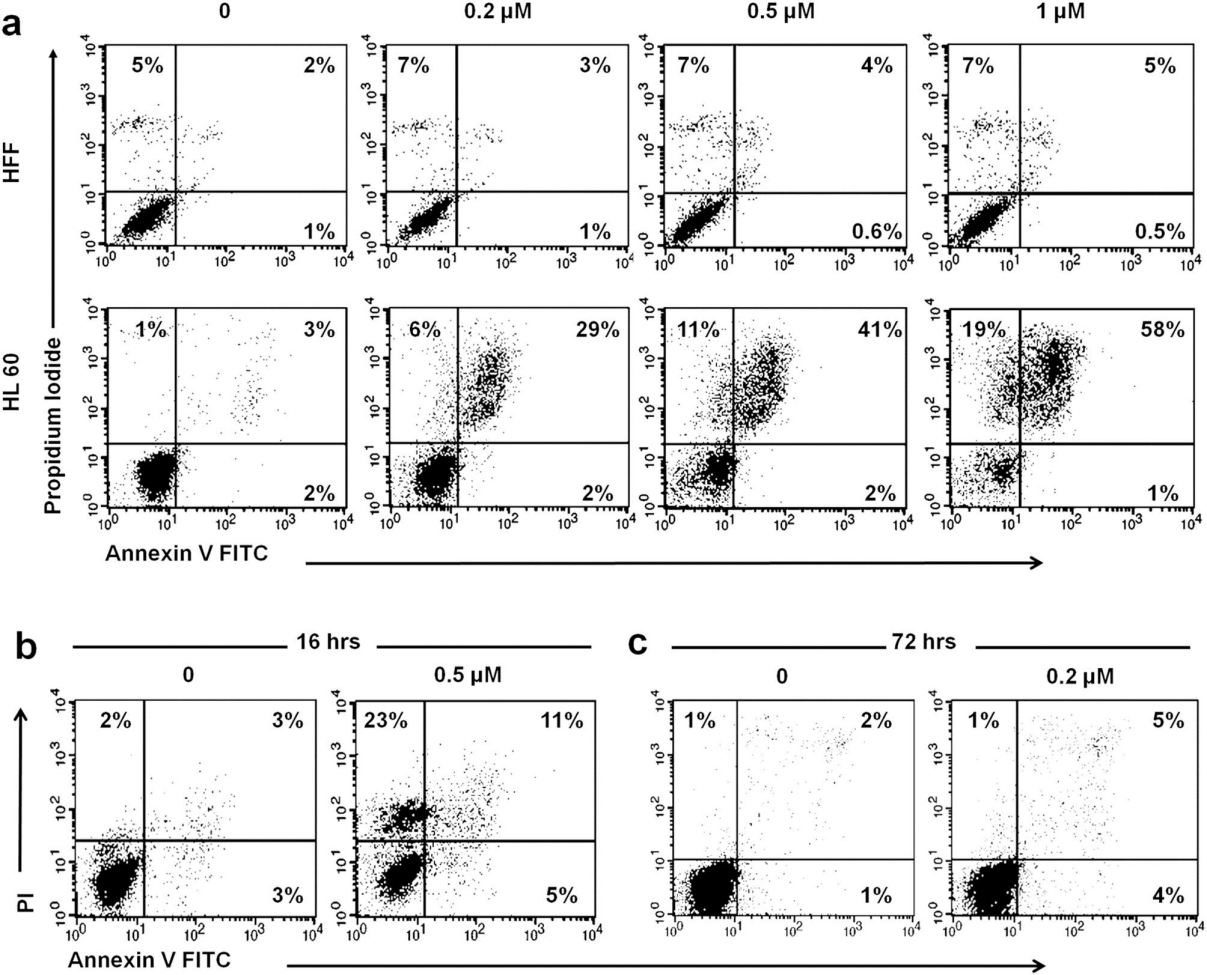
Flow cytometry analysis of fenbendazole treated cells with Annexin V-FITC: (**a**) concentration dependent selective cell death induced in HL60 leukaemia cells by fenbendazole compared to Human Foreskin Fibroblast cells (HFF); (**b**) 16 h post 0.5 µM fenbendazole treatment (16% apoptotic (Annexin+) and 23% necrotic cells (Annexin−, PI+)); (**c**) 72 h post 0.2 µM of fenbendazole (8% apoptotic (Annexin+) and 2% necrotic cells (Annexin−, PI+)). It can be seen that longer time point, and less concentration increases percentage of apoptotic cells over necrotic one.

In the next step, the nature of cell death and selectivity of the compound was further investigated. Flow cytometry analysis of cells treated for 24 h with fenbendazole via Annexin V-FITC demonstrated a concentration-dependent selective cell death induced in leukaemia cells, around the LC50 of the compound, with no significant effect on normal HFF cell population (Fig. 4a). Further experiments revealed that in a shorter time point (16 h) post 0.5 µM fenbendazole treatment, necrotic cells (23%) outnumbered apoptotic cells (16%, see Fig. 4b), while after incubating the HL60 cells with 0.2 µM fenbendazole for 72 h apoptotic cells (8%) outnumbered necrotic cells (2%, Fig. 4c). Based on these findings it was observed that higher concentrations of fenbendazole lead cells to a sudden death, which much resembles necrosis, while lower concentrations convert cells to granulocytes and subsequently induce programmed cell death, in agreement with the previous findings from microscopy.

Although some derivatives of benzimidazole anthelmintics, such as mebendazole, albendazole, and flubendazole, draw increasing attention as potent anticancer agents^28^, to the best of our knowledge, fenbendazole has not been previously used in the treatment of leukaemia. It has however been tested against other cancer types where it was found to induce apoptosis in a lung cancer cell line by accumulating apoptosis regulatory proteins such as cyclins, tumour proteins p53 (*TP53*), and IĸBα and induced stress-associated genes like *HSPA5*(*GRP78*), *DDIT3*(*GADD153*), *ATF3*, *ERN1* (*IRE1α*) and *PMAIP1*(*NOXA*)^29^. Fenbendazole alone was previously reported to be ineffective in a lymphoma mice xenograft model, but efficacious when co-administered with a vitamin-supplemented diet^30^. The highest dose of fenbendazole did not change the growth of mammary tumours (EMT6 cell line) or radiation efficacy in one study^31^, and did not alter the dose–response curves when combined with Docetaxel^31^.

### Analysis of the mode-of-action of fenbendazole

As mentioned before, the mode-of-action of a compound can be considered on a systems level, e.g. via the induced gene expression changes, or on a protein level, both approaches of which have significant strength in different areas (most notably when it comes to efficacy in the former case, and understanding ligand–protein interactions, and hence supporting lead optimization, in the latter case). We now aimed to understand the mode-of-action of fenbendazole on both a systems level, and a protein level, in agreement with the whole algorithmic approach presented in this work.

In silico mode-of-action prediction^11^ suggests that on the protein level, fenbendazole targets the Angiopoietin-1 receptor (TIE-2), Aurora kinase B, RAF proto-oncogene serine/threonine-protein kinase (RAF1) and Vascular endothelial growth factor receptor 2 (VEGFR2), as well as Aurora kinase A (Table 1). Aurora kinase B has been previously suggested to be a promising therapeutic target for leukaemia^32^. RAF1 is important in inducing apoptosis in leukaemia cells and is related to relapse-free survival of AML patients^33,34^. Also on a compound-set level in silico target prediction identified RAF1 as the most enriched targeted protein among the top 50 compounds (with a P value of 0.0016, see Table 2). The Comparative Toxicogenomics Database (CTD)^17^ also identified relatively high scores (14, 20, 21, 25, 12, with an average of 18) for these predicted protein targets in leukaemia, agreeing with our predictions. The average of the five top target scores of terazosin and proxymetacaine were lower (5 and 4 retrospectively), indicating that there is more evidence linking fenbendazole to this indication, compared to terazosin and proxymetacaine, based on both bioinformatics and cheminformatics information.

**Table 2:** Most enriched protein targets in the top results.

Target ID	Target name	Gene symbol	Count in top 50 predicted compounds	Count in all CMap compounds	Probability	Average position of the target	Average rank	P value
CHEMBL1906	Serine/threonine-protein kinase RAF	RAF1	4	61	0.066	4.5	10.5	0.00165
CHEMBL4308	Bradykinin B1 receptor	BDKRB1	4	62	0.065	5.3	21.3	0.00175
CHEMBL4081	Coagulation factor III	F3	5	116	0.043	2.6	11.2	0.00267
CHEMBL210	Beta-2 adrenergic receptor	ADRB2	7	279	0.025	1.4	26.3	0.00716
CHEMBL213	Beta-1 adrenergic receptor	ADRB1	7	283	0.025	3.0	26.3	0.00768
CHEMBL1941	Histamine H2 receptor	HRH2	4	97	0.041	5.5	31.0	0.00819
CHEMBL246	Beta-3 adrenergic receptor	ADRB3	7	337	0.021	2.7	26.3	0.01720
CHEMBL2056	Dopamine D1 receptor	DRD1	7	369	0.019	2.6	27.6	0.02529
CHEMBL234	Dopamine D3 receptor	DRD3	6	296	0.020	5.0	31.2	0.02833
CHEMBL1821	Muscarinic acetylcholine receptor M4	CHRM4	5	220	0.023	5.6	27.2	0.02944
CHEMBL2035	Muscarinic acetylcholine receptor M5	CHRM5	4	194	0.021	4.3	29.5	0.06092
CHEMBL217	Dopamine D2 receptor	DRD2	6	416	0.014	3.0	30.8	0.08312
CHEMBL231	Histamine H1 receptor	HRH1	6	450	0.013	1.3	33.3	0.10076

Frequency of all predicted protein targets were counted in the top 50 results compared to all compounds in the Connectivity map database. Probability of observing each protein target (count in top 50/count in all compounds) is displayed as Probability. P value displays significance of observing each target in the top results compared to all the compound signatures extracted from CMap (5,764 signatures). Average position of the target denotes average rank of the protein target for the top 50 compounds using *in* silico target prediction.

The cheminformatics approach enabled us to select fenbendazole over mebendazole due to its more relevant predicted bioactivity profile. The bioactivity profile predicted for both fenbendazole and mebendazole included Tyrosine-protein kinase TIE-2, Serine/threonine-protein kinase RAF and Vascular endothelial growth factor receptor 2. However, fenbendazole’s predicted bioactivity profile only included Serine/threonine-protein kinase Aurora-A and B. It is known that inhibition of Aura A and B^32^ induces apoptosis in leukaemia AML cells^35^. Also, Aurora kinase A is required for hematopoiesis^36^ and its expression is increased in leukaemia stem cells^37^. This advantage on the cheminformatics side enabled us to select fenbendazole over mebendazole (Table 1).

The GSEA algorithm also identified the genes most upregulated by fenbendazole treatment of HL60 cells (instance ID 2360), which are in turn downregulated in the disease, to be *RGS2*, *FPR1*, *SAT1*, *PLSCR1*, *PTPRE*, *FCER1G* and *CD55* (Supplementary Table S1). Among those *RGS2* showed highest upregulation in the compound signature, and literature shows that this gene is involved in myeloid differentiation and leukemic transformation, thereby providing a biological rationale for compound selection^38^. *SAT1* is overexpressed in intestinal cancer, breast cancer and melanomas, compared to control^39^, while *PLSCR1* has been previously linked with proliferation arrest of leukaemia cells and granulocyte-like differentiation, as well as causing downregulation of *MYC*, which is also implicated in the latter process (as discussed below)^40^. Also the upregulation of other genes has been mechanistically liked to the development and progression of leukaemia (or other cancers): protein phosphatases including *PTPRE* are important regulators of cell signalling and their deregulation contribution to cell transformation^41^, while expression of *PTPRE* is reported to be significantly upregulated in AML leukaemia itself^41^, and *FCER1G* is significantly downregulated in CML leukaemia patients where it is also associated with T-cell immunodeficiency^42^. On the other hand, fenbendazole treatment on HL60 cells *downregulates WDR12*, *MYC*, *WDR3*, *CTH*, *TTC27* and *ATF5*, which are in turn significantly upregulated in the disease state. Here, *WDR12*, *MYC*, *WDR3*, are critical in the regulation of cell cycle progression in cancer cells^43,44^. MYC is important in cell cycle progression and transformation as well as induction of apoptosis (http://www.ncbi.nlm.nih.gov/gene/4609) and it has been associated with the differentiation of HL60 cells to granulocyte^24,45^. Tretinoin (ranked 15) and genistein (ranked 16) as known inducers of differentiation of HL60 cells also downregulate *MYC. ATF5* is widely expressed in carcinomas and has previously been shown to be a selective target for breast cancer treatment^46^. On the pathway level, fenbendazole is predicted to target the “PI3K-Akt signalling” pathway, which is known to be active in acute myeloid leukaemia^47^, by inhibiting TIE-2, RAF-1 and VEGF and downregulating the Myc proto-oncogene protein (MYC), while upregulating cyclin-D3 (CCND3). The “Cell Cycle” pathway is also enriched by inhibiting Aurora kinase-B and Aurora kinase-A and downregulating *TUBB*, *TUBB4B*, *MYC* and *TUBA1A* and upregulating *CCND3*. Another important enriched pathway is the “Acute myeloid leukaemia” pathway by inhibiting *RAF1* and down-regulating *MYC*. We can hence conclude that genes targeted by fenbendazole according to CMap data has biological relevance to leukaemia according to literature.

**Figure 5:**
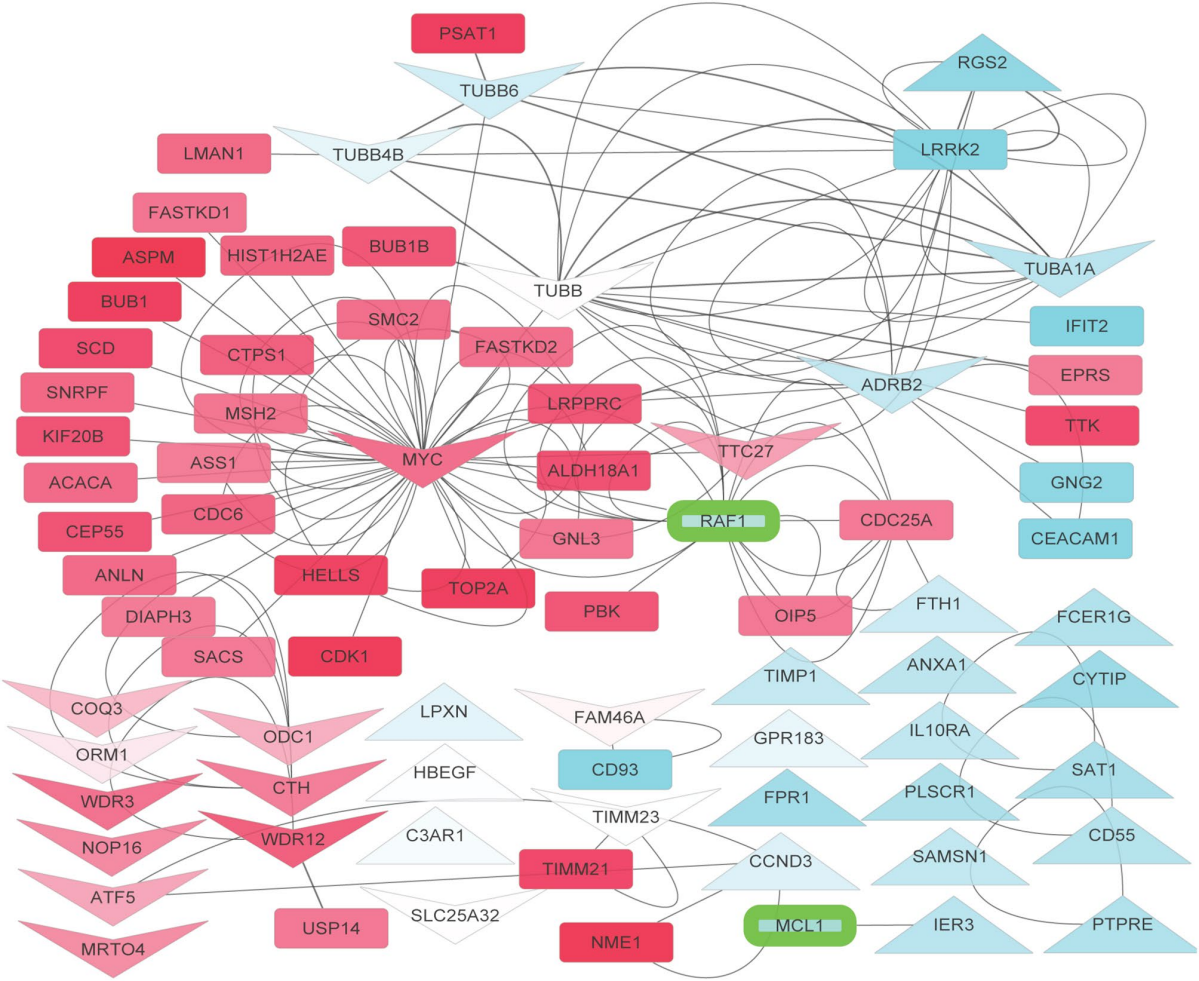
Protein–protein interaction network of the proteins associated to fenbendazole-induced gene expression changes and its protein targets: each node represents a gene specified by the gene symbol, arrows indicate up- and downregulation of genes by fenbendazole (according to CMap data) and the rest of genes are in a rectangle. The genes that were up/down regulated in leukaemia disease signature are highlighted in red and blue, respectively. Predicted protein targets of fenbendazole are highlighted with green border. *MYC,* transcription factor that plays a role in cell cycle progression, apoptosis and cellular transformation, is a key gene in the topology of this network; it shows highest ‘betweenness’ in the connected part of the network. *MYC* is upregulated in the disease signature and in turn is downregulated by fenbendazole. On the other hand, RAF1, which is predicted as a target from the cheminformatics-side, is a first neighbour of many genes dysregulated in leukaemia, indicating the multi-faceted nature of the mode-of-action of a compound.

Based on the above information, we next attempted to understand the mode-of-action of fenbendazole with a network analysis approach. Figure 5 depicts the differentially expressed genes in leukaemia as well as gene targets (from CMap) and the predicted protein targets of fenbendazole. In agreement to the above GSEA approach, the network approach points out the particular importance of *MYC*. It appears that *MYC* has a particular importance in leukaemia where it is significantly upregulated (six folds), while at the same time being connected to 23 other upregulated genes and having highest ‘betweenness’ (0.87) of the most connected part of the network. It is known that *MYC* regulates several other genes involved in growth, cell cycle, signalling, and adhesion^48^ which is in agreement with the network visualization. fenbendazole targets *MYC* by downregulating it and based on all information available this seems to be of major relevance for its mode-of-action. One of the likely supportive activities of fenbendazole is the predicted inhibition of RAF kinase, which has a high ‘betweenness’ (ranked third for betweenness, 0.17) in the network and its indirect inhibition appears to lead to efficient disruption of the network. Even though *RAF* is not significantly differentially expressed in the leukaemia signature itself, it is the first neighbour of significantly differentially expressed genes of the disease, such as *PBK, OIP5* and *CDC25A*, and may thereby exert indirect effects on the system. In agreement with the above discussion, The importance of *RAF1* has been previously established for leukaemia^33,34^. *TUBB* (ranked second for betweenness, 0.26), *TUBA1A, TUBB6* and *TUBB4B* are also similarly important as they have high betweenness and connected to differentially expressed genes of *TTK, EPRS, PSAT1, LMAN1* and *BUB1B* even though they are not differentially expressed themselves.

Furthermore, we explored whether pathway annotations such as Gene Ontology (GO) would add information to understanding the mode-of-action of fenbendazole. The network analysis suggests that fenbendazole enriches the rather broad “negative regulation of biological process” (GOID: 48519) by upregulating *HBEGF, TIMP1, ANXA1, PTPRE, FTH1, RGS2, IER3* and downregulating *CTH, ATF5, MYC, ADRB2* and inhibiting RAF1 and MCL1 proteins as suggested by the target prediction algorithm. More specific in the current context, the compound enriches the “Apoptosis” pathway by inhibiting *RAF1* and *MCL1* and “natural killer mediated cytotoxicity” by downregulating *TUBB* and *TUBB4B*. *CTH,* which is downregulated by fenbendazole, is also a member of the “negative regulation of apoptotic signalling” pathway (GO: 2001234). The experimental validation of the effects of fenbendazole to induce differentiation followed by programmed cell death is hence consistent with the enriched biological processes from this section of the work.

### Experimental validation of the mechanism of fenbendazole

**Figure 6:**
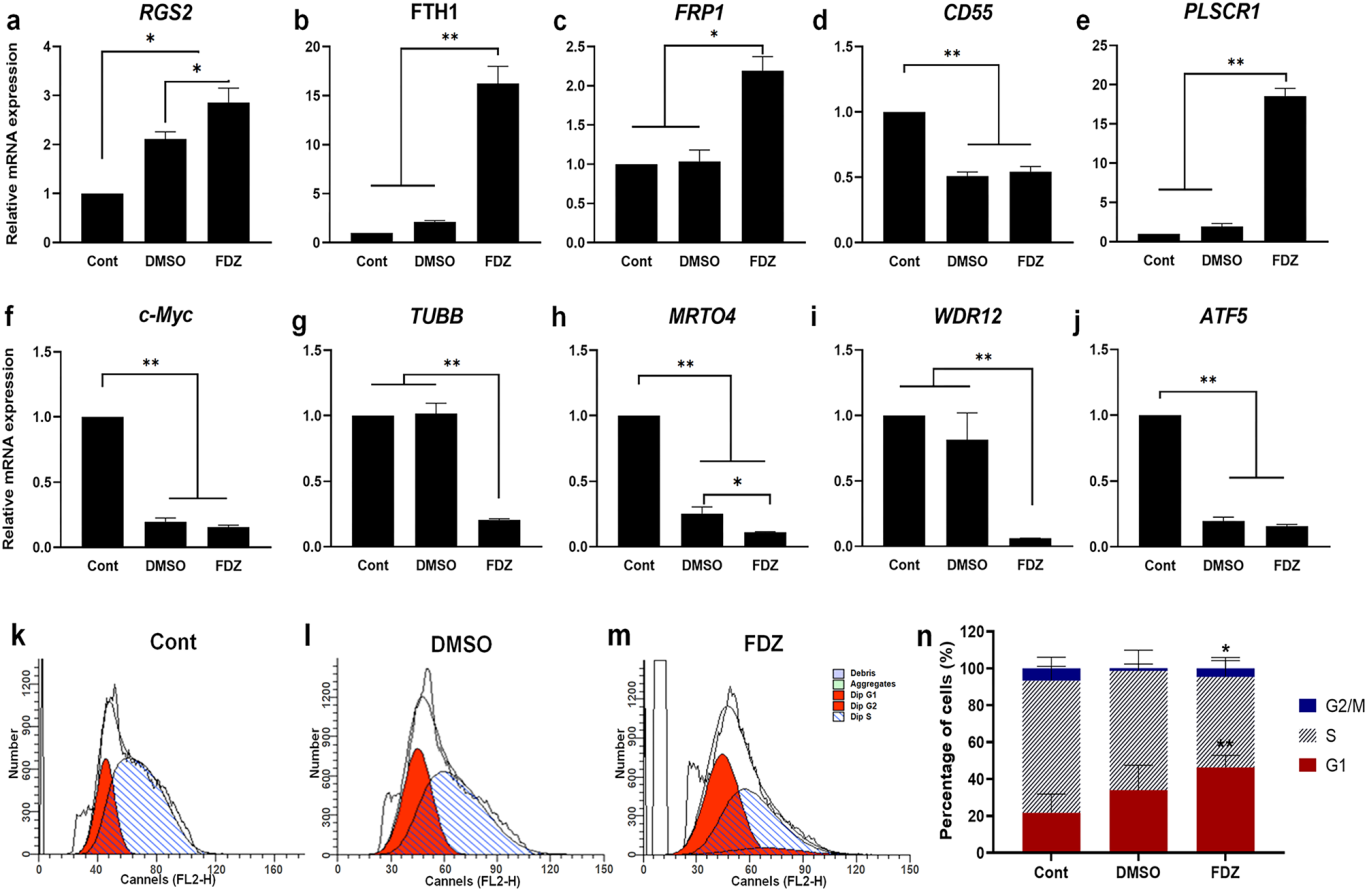
Mode-of-action analysis of fenbendazole (compared to DMSO control and relative to HL60 cells prior to treatment). The relative RNA expression level of genes expected to undergo upregulation after treatment according to our model: (**a**) *RGS2*, (**b**) *FTH1*, (**c**) *FRP1*, (**d**) *CD55*, and (**e**) *PLSCR1* as well as downregulation: (**f**) *c-Myc*, (**g**) *TUBB*, (**h**) *MRTO4*, (**i**) *WDR12*, and (**j**) *ATF5*. Representative histograms of cell cycle analysis of (**k**) Control, (**l**) DMSO-, and (**m**) fenbendazole- treated HL-60 cells, and (**n**) number of cells per cell cycle phase following DMSO and fenbendazole treatment. *P < 0.05 and **P < 0.01. Each value is presented as mean ± SEM of three independent experiments.

Expression of few up/down regulated genes in the fenbendazole signature extracted from CMap were confirmed experimentally in this work after longer time point. The fenbendazole instance from CMAP was at dose 13 µM after 6 h treatment on HL60 cells (instance ID: 2360). Here we check those genes at longer timepoint of 5 days in mRNA level. Among up-regulated genes in response to fenbendazole, the expression of *RGS2*, *FPR1*, *FTH1*, *PLSCR1*, and *CD55* was assessed after 5 days of treatment with fenbendazole as well as DMSO, which were normalized to the expression level in cells prior to induction (see Fig. 6a–e). It can be seen that all genes except CD55 were significantly upregulated as the result of fenbendazole treatment with the higher level, in comparison to DMSO treated cells.

We experimentally checked RNA expression of PI3K-Akt signalling genes to investigate effect of fenbendazole in this pathway which was in agreement with pathways enriched based on fenbendazole signature in CMap data. We found a remarkable decline in the expression of MYC, MRTO4, and ATF5 due to the induction by either fenbendazole or DMSO (Fig. 6f,h,j). On the other hand, significant down regulation occurred for TUBB and WDR12 only following fenbendazole treatment (Fig. 6g,i). To determine whether fenbendazole treatment is accompanied with the accumulation of cells in the non-division phase, cell cycle analysis was carried out, using propidium iodide (PI) staining^49^. As expected, fenbendazole triggered significant G1-phase cell cycle arrest in HL60 cells, mainly via decreasing of cell population in S phase (Fig. 6k–n). However, DMSO treatment slightly, but not significantly, increased cells in G1 phase from 22% in control to 34%.

**Figure 7:**
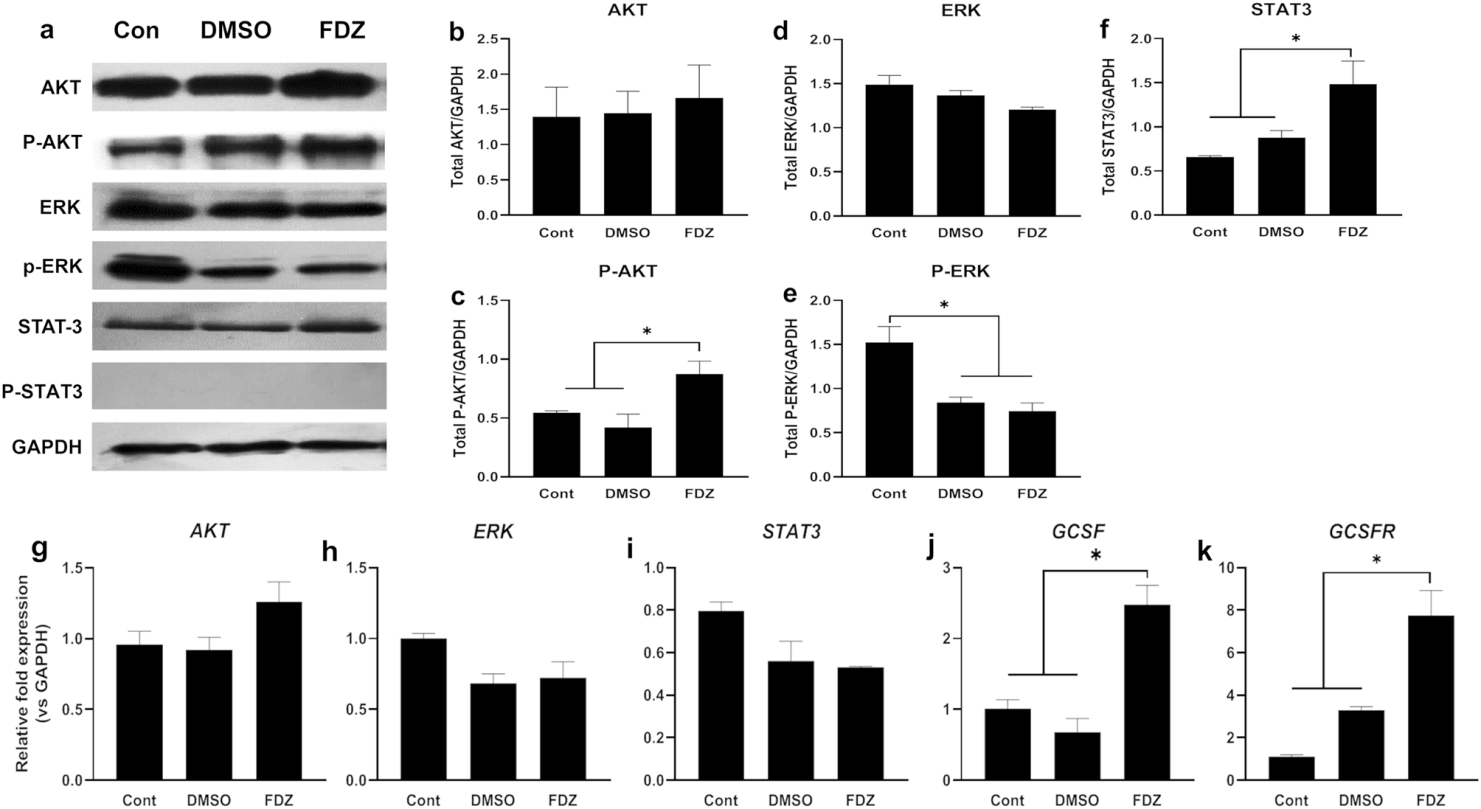
Pathway analysis of HL-60 cells treated with DMSO and fenbendazole determined by Western Blotting. (**a**) Expression and phosphorylation levels of AKT, ERK, and STAT3; the quantitative analysis of relative protein level of (**b**) Total AKT, (**c**) p-AKT, (**d**) Total ERK, (**e**) p-ERK (**f**) Total STAT3 vs GAPDH; and the relative RNA expression level of (**g**) AKT, (**h**) ERK, (**i**) STAT3, (**j**) GCSF, and (**k**) GCSFR following fenbendazole and DMSO (control) treatment, compared to HL-60 cells prior treatment (day 0). *P < 0.05 and **P < 0.01. Each value is presented as mean ± SEM of three independent experiments. As the blots were cropped, full length blots are provided in Supplementary Figure S1.

In order to investigate the role of PI3K/AKT, MEK/ERK or JAK/STAT pathways in the induction of neutrophil differentiation in HL-60 cells by DMSO or fenbendazole, the activation of key effector proteins of these signalling pathways were evaluated through Western blot analysis. Figure 7a displays the expression level of total AKT, ERK1/2, and STAT3 as well as the amount of the phosphorylated form of each protein. Densitometric quantification of bands relative to GAPDH showed a slight, but not significant increase in total AKT for fenbendazole and DMSO treated samples (Fig. 7b,c). However, only fenbendazole treatment (but not DMSO) resulted in a significant increase in phosphorylation of Ser-473 of AKT. In contrast, level of ERK and p-ERK were reduced in response to both interventions, which was specifically effected by ERK activation (Fig. 7d,e). In addition, total expression level of STAT3 was highly elevated in response to fenbendazole (Fig. 7f). However, the activated p-STAT3 (Tyr-705) was not detected in HL60 cells in either DMSO- and fenbendazole-treated groups. We also found that the mRNA expression of AKT and ERK were showed almost similar pattern as their proteins (Fig. 7g,h). The mRNA level of STAT3 significantly declined after application of both fenbendazole and DMSO (Fig. 7i). These were accompanied with a marked rise in the expression of GCSF and GSFR mRNA, following fenbendazole treatment (Fig. 7j,k).

Although several studies have highlighted the role of STAT3 activity in myeloma cell lines^50,51^, there is controversy about its importance in HL60 cells^52,53^. According to the undetectable level of p-STAT3 in all groups, the regulatory effect of JAK/STAT pathway in this process requires further investigation. Notably, HL-60 as a cancer cell line has several new mutations over time and exhibits genetic/epigenetic heterogeneity in cells provided from different sources^54^. Furthermore, the mRNA expression of *STAT3* did not show any significant changes in response to fenbendazole treatment, however, total STAT3 proteins after fenbendazole treatment is significantly higher. On the other hand, there have been partially contradictory findings on the involvement of the MAPK/ERK pathway on the maturation of granulocyte progenitors. While some studies support the proliferation effect of this molecule^55^, other studies approve its role in differentiation^56,57^. Concerning this, we provide some evidence that DMSO, independent of fenbendazole, significantly decreased ERK expression in both mRNA and protein level.

## Discussion

In this work, we have employed an integrated transcriptional drug repositioning and cheminformatics approach to find novel small molecules that can induce differentiation of leukaemia cells to granulocytes. The approach rediscovered current standard of care, retinoic acid, for APL (HL60 cells) that is a well-known drug for differentiation therapy along with some novel candidates. Among highly ranked compounds, fenbendazole was shortlisted as a suitable candidate and validated experimentally that it can induce differentiation of HL60 cells to granulocytes. Moreover, it was shown that the compound exhibits 14.5-fold selectivity in killing HL60 leukaemia cells at low doses of 0.36 µM and 0.31 µM at 48 and 72 h compared to bone marrow stem cells (5.1 µM and 4.5 µM after 48 and 72 h). This confirms that the compounds that induce the differentiation are generally less toxic than chemotherapy agents^3^.

In this work, we have also investigated the mechanisms underlying neutrophilic differentiation induced by fenbendazole via studying three major pathways underlying granulocyte differentiation, including PI3K/AKT, JAK/STAT and, MAPK pathways. To this end, we studied expression and activation of key effector proteins of mentioned pathways. Our results showed that following induction of neutrophil differentiation, expression of AKT in both mRNA and protein level, have not significantly changed, thus it seems that this process was induced independent of AKT expression. Whereas AKT activation (via ser-473 phosphorylation) elevated during fenbendazole treatment and this result occurred independent of DMSO, as vehicle. PI3K/AKT pathway is known as an important regulator of various physiological events such as apoptosis, progression of cell cycle, differentiation, and metabolism. Notably, up-regulation of this pathway can be detected in majority of cancers and facilitates tumour growth, angiogenesis and therapy resistance^58^. Furthermore, constitutive activation of PI3K/AKT signalling has been known as a common event in AML patients^59^. Therefore, efficient blocking of PI3/AKT pathway, seems to be a potent regulator to inhibit proliferation of cancerous cells, especially in AML. Despite the mentioned overall role of this pathway, activation of AKT induced by G-CSF resulted in differentiation, but not proliferation, in myeloid precursor cells^60^. Also activation of this pathway has been reported during granulocytic differentiation of HL_60 and NB4 cell lines following ATRA induction^61,62^. To elucidate the potential impact of GCSF on AKT activation, the effect of fenbendazole on G-CSF signalling, G-CSF and G-CSFR expression were measured in mRNA level, which showed remarkable overexpression of both genes. G-CSF is known as a hematopoietic cytokine, which have critical role on neutrophil progenitors survival and stimulates them in bone marrow to proliferate and differentiate to functional neutrophils^63,64^. It is known that G-CSF receptor show highest level of expression in neutrophils^65^. In addition, various mutations in the *CSF3R* have been reported in myeloid disorders including, chronic neutrophilic leukaemia (CNL), myelodysplastic syndrome (MDS), Acute Myeloid Leukaemia (AML), and atypical chronic myelogenous leukaemia (aCML)^55^, which confirmed the importance of this receptor in neutrophil differentiation. GCSF cytokine binds to extra cellular domain of GCSFR and through its cytoplasmic domain, activates two major protein tyrosine kinases: SRC family kinase and Janus kinase which effect on multiple intracellular signalling cascades. During GCSF stimulation, SRC kinase activates AKT protein in a separate mechanism from STAT and ERK. Mediating PI3K protein and JAK kinase likely have major role on activation of STAT^66^. So, it seems that the impact of fenbendazole, at least partially, is associated with activation of GCSF/GCSFR signalling, which increased AKT activity through SRC cascade.

In this work, we highlight one important application of drug repositioning approaches in differentiation therapy and how our combined bioinformatics and cheminformatics approaches can facilitate selection of small molecules for this purpose. We also show that the choice of disease signature and the two states that are being compared is directly influencing the biological outcome we would want to achieve. As opposed to conventional approaches of drug repositioning, the comparison does not have to necessarily be between disease and healthy states. It should be viewed as comparison of two biological states. One representing the current state and one as a target state. Here we have shown using a differential transcriptional profile between HL60 cells with granulocytes serves as a blueprint for selecting compounds that induce dedifferentiation of cell types, of relevance for cell differentiation therapy in leukaemia. After identifying the right signature for differentiation, the challenging part is to prioritise compounds. The Bioinformatics scoring system rank ordered all drug signatures in the CMap database from 1 to 5,765. Then, the list was filtered to the top 30 drugs. For prioritisation, we took into account the cheminformatics scoring system that scores compounds based on the predicted targets and their relevance to leukaemia. These two scoring systems accompanied with novelty literature search only prioritised one single novel drug, fenbendazole, with good scores in bioinformatics and cheminformatics scoring system. The Network visualisation approach discussed, facilitated elucidating mode of action of selected compound candidate and enabled visualising all potential targets of a candidate drug and how it is placed in the protein–protein interaction network of differentially expressed genes in the leukaemia differentiation signature. All bioinformatics, cheminformatics and network approaches are useful for shortlisting compounds and after that further literature search might be useful for selecting compounds for experimental validation.

The authors have also shown previously that comparing stem cell and cardiomyocyte gene expression profiles can lead to blueprint of differentiation of stem cells to cardiomyocyte and compounds that come up out of the drug repositioning can cause the differentiation of stem cell to cardiomyocytes^67^. This highlights a new way of observing transcriptional drug repositioning approaches in general and how its application can facilitate identifying small molecules for differentiation therapy and beyond.

## Online methods

### Pre-processing of CMap

CMap^8^ provides a rank matrix of all genes for all compound instances. This data was used for the generation of rank-ordered list of compounds for breast cancer (GDS2626). In case of leukaemia and large-scale diseases, raw CEL files of CMap were preprocessed. For this purpose, CEL files were obtained from the CMap website and Factor Analysis for Robust Microarray Summarization (FARMS)^68^ was utilised in R to preprocess the cell files. The data consisted of three cell lines (MCF7, PC3, and HL60) and three different array types (HGU133A, HTHGU133A, and EA.HTHGU133A). Different combinations of array types and cell types were pre-processed separately. Custom CDF definitions (from Brainarray) were utilized and then I/NI filtering^69^ was performed, using the informative (I)/non-informative (NI) calls approach integrated in FARMS. This allows filtering out genes for which the probes did not show a consistent behaviour across different samples. FARMS was performed with a Laplacian prior with default settings in order to keep even the least informative genes.

Log fold changes of gene expression were calculated by dividing the intensity value for each gene by the intensity value of the respective vehicle. When multiple vehicles were present, the vehicle closest to the spatial median of all vehicles was chosen. The spatial median was preferred over the standard median because it maintains the correlational structure of the genes of each sample.

### Transcriptional drug repositioning approach

For identifying compounds and diseases correlated and anti-correlated in gene expression space, Gene Set Enrichment Analysis (GSEA) as implemented by the Broad Institute^16^ was employed in this work. This method checks whether a query gene signature is occurring at the extremes (top or bottom) of a rank-ordered list of genes, or whether it shows closer to random distribution (i.e., there is no correlation between both spaces). An enrichment score was calculated by descending the rank-ordered list of genes and incrementing a variable when encountering a gene in the given query signature. The magnitude of increment depends on the position of the gene in the list, which is chosen corresponding to a weighted Kolmogorov–Smirnov statistic. The enrichment score can range between − 1 and 1, where − 1 shows strong negative connectivity, 1 identifies strong positive connectivity and 0 represents zero connectivity. The query signature in the GSEA approach is a short list of differentially expressed genes of a disease, which are used to search the full rank-ordered gene expression profile of drugs. However, in the current work, the query signature was chosen to be the differentially expressed genes caused by compound treatment, which were screened against the full gene expression profile of a disease. The reason for this choice was that the disease signal was generally found to be stronger than the gene expression signal after compound treatments. In addition, due to the noisy nature and low fold-changes of gene expression data from compound treatments, only the 20 most over- and under-expressed genes were employed. The optimal cut-offs were chosen after using various different cut-offs and selecting the one with maximum precision (percentage of predicted compounds in top results which were supported by literature) for leukaemia. For each compound, two query signatures, namely one of the most upregulated genes ($scor{e}_{up}$), and one of the most downregulated genes ($scor{e}_{down}$) were used. For each disease vs. drug the following formula was used to combine the two scores:

$$score=\frac{scor{e}_{up}-scor{e}_{down}}{2}$$

The combined score was used to rank order the compounds in CMap database for the leukaemia differentiation gene expression profile.

### Cheminformatics approach

A target prediction algorithm as established before^11^ has been utilised to predict protein targets of compounds in the CMap databases using the Naïve Bayes approach. This algorithm predicts a score for each protein target included in the training set, which represents the probability of the compound ability to bind to this target (without considering the nature of the particular effect, say agonism, antagonism, etc.). The extraction of compound-target pairs was identical to the benchmarking dataset query introduced in the previous work^11^ (which included targets with binding affinity less than 10 µM and confidence level of 9 or 10) except that it was applied on ChEMBL^19^ v.17 and hence left us with a training database of 385,126 compound-protein pairs, 1643 distinct proteins and 226,791 unique compounds. Compounds were standardised and ECFP4 fingerprints were generated using the JChem package of ChemAxon (Jchem 6.1.2. *ChemAxon, *http://www.chemaxon.com, 2013). The standardisation options were Aromatise, RemoveExplicitH, Clean 2D, Clean 3D, RemoveFragment and Neutralise. The Laplacian modified Naïve Bayes version of the algorithm provided in the previous publication^11^ was then trained on the extracted data.

The cheminformatics part of the integrated approach includes prediction of targets for all compounds. In order to identify the importance of predicted targets for a disease of interest, the Comparative Toxicogenomics Database (CTD)^17^ has been used. Disease-gene links were downloaded separately as provided on the CTD website. The CTD database provides an inference score for each target which indicates the level of relevance of each target with each disease, based on text mining approaches of a large set of scientific publications. The gene-protein links were retrieved from ChEMBL^19^ to map gene identifiers to proteins implicated in diseases.

A cheminformatics score for all compounds in CMap was calculated. This involved averaging relevance score to leukaemia (Extracted from CTD) for top seven protein targets predicted (using the in-silico approach) for each of the compounds in the connectivity map database.

### Network visualization method

The list of up/downregulated genes of fenbendazole Instance in CMap (instance-ID 2360) as well as protein targets predicted with the target prediction algorithm^11^ was prepared. This list was searched in Cytoscape^70^ public network databases and the proteins-protein interactions of those genes were retrieved from the Mentha database^71^. This yields a visualisation of proteins as nodes and their interactions as undirected edges. The nodes in Cytoscape are linked to a table carrying all the information of the protein including the protein Uniprot ID, Gene Symbol and Entrez Gene ID. Gene expression data of leukaemia was retrieved from the GEO database GSE48558, where HL60 cells were compared to granulocytes. GenePattern^72^ was used to pre-process the leukaemia database and following this log fold changes of the HL60 samples over granulocytes were calculated. In order to load this information to Cytoscape, it was required to map probe IDs to gene ID/Uniprot ID. Hence, the probe IDs of the array which were used in the leukaemia database (Affymetrix Human Gene 1.0 ST Array, GPL6244) were mapped to Entrez Gene IDs, Uniprot IDs and Chembl IDs using the Ensembl Biomart interface. All the information (gene expression changes and gene mappings) were loaded in to Cytoscape. As a result, the log-fold changes of each gene were represented in each node of the network. Then the genes with log-fold change above 1 or below − 1 were selected and exported to a new smaller network to take closer look at the most important genes involved in leukaemia, as well as their interactions. The nodes were then coloured relative to their gene expression log fold change for better representation of the leukaemia protein interaction network. Then it was aimed to see which of these genes are targeted by the predicted compound (fenbendazole). For this purpose the list of up and downregulated genes of the compound from the CMAP data (instance 2360 applied on HL60) was achieved and loaded into Cytoscape. Up/down genes were visualized with up and down arrows on the respective nodes of the network. Furthermore, the predicted proteins that were inhibited by the compound were also loaded to the node information and visualized in with a larger size and green border colour. Up/downregulated genes and inhibited proteins were selected using the filter tool. The BINGO plugin^73^ was utilized to calculate pathway enrichment using the Fisher exact test and Homo sapiens GO biological processes.

### Culture methods for drug toxicity measurement

Human Bone Marrow-derived mesenchymal Stem Cells (BMSC) were kindly gifted by Royan cell therapy centre and Human Foreskin Fibroblast cells (HFF) were isolated from human foreskin as previously described following obtaining informed consent form volunteer donors^74^. HL60 promyelocytic leukaemia cell lines were purchased from the Iranian National Cell Bank (Pasteur Institute of Iran, Tehran). BMSC and HFF cells were cultured in DMEM/F12 medium, supplemented with 10% (v/v) heat inactivated fetal bovine serum (FBS), 1% (v/v) NEAA, 1% (v/v) l-glutamine, 1% (v/v) Penicillin/Streptomycin, and incubated at 37 °C in a humidified 5% CO_2_ incubator. Human leukaemia HL60 cells were maintained in RPMI 1640, supplemented with 1% (v/v) l-glutamine 10% (v/v) heat inactivated fetal bovine serum (FBS), 1% (v/v) NEAA, 1% (v/v) Penicillin/Streptomycin (all from Gibco, Paisley, UK), and incubated at 37 °C in a humidified 5% CO_2_ incubator. HL60, BMSC and HFF cells were plated according to their growth curves with 100,000, 35,000 and 30,000 cells respectively in each of 24 wells of cell culture plates. One hour later, serial concentrations of each predicted compound (5 µl/well) were added to cultured cells with equal amounts, based on literature data. To serve as solvent control, appropriate solvent concentrations, as the one used for maximum concentration of drugs were added to untreated wells. Cells were treated for 24, 48 and 72 h and then 50 µl MTS solution (Promega, WI, USA) was added to each well. Absorbance was measured 3.5 h later at 450 nm using an ELISA microplate reader (Stat fax 3200, Awareness Technology Inc.). Cell viability was calculated using the following formula: cell viability (%) = (mean experimental absorbance/mean control absorbance) × 100 and presented as means ± SDs.

### Verifying differentiation of leukaemia cells to granulocytes

We subsequently asked whether or not the fenbendazole treated HL60 cells were converted to neutrophil granulocytes. The cells were cultured for 1–6 days with 0, 0.1 µM, 0.2 µM and 0.5 µM of fenbendazole and 1.25% Dimethyl sulfoxide (DMSO) as a positive control (fenbendazole was dissolved in 0.65% DMSO). To evaluate the morphological features of treated cells, they were subjected to Wright-Giemsa staining following smear preparation and methanol fixation, as described earlier^75^. Cell transformation was also evaluated using the Nitro Blue Tetrazolium (NBT) reduction assay. The cells were resuspended in RPMI-1640 media containing 1 mg/ml of NBT and incubated at 37 °C in 5% CO_2_ for 25 min. Afterwards, the blue formazan particles were dissolved in DMSO and 2 M potassium hydroxide and their absorbance at 630 nm was determined.

### Finding the nature of cell death

In order to study the nature of cell death induced by fenbendazole, cells from two HL60 and HFF cell lines were incubated with various concentrations (0 µM, 0.2 µM, 0.5 µM and 1 µM) for 24 h and apoptosis was analyzed using an Annexin-V Apoptosis Detection kit (IQP-116F) with a FACSCalibur Flow Cytometer (Becton Dickinson, San Jose, CA, USA). Briefly, according to the modified manufacturer's instruction, 10^6^ cells were collected by centrifugation (1500 rpm, 10 min), washed in calcium buffer and resuspended in Annexin V staining buffer. The cells were then counterstained with Propidium Iodide (PI), and finally treated with 50 μg/ml RNase A for 15 min at 37 °C. We also examined the apoptotic effect of 0.5 µM fenbendazole both after 16 and 24 h of treatment using the abovementioned procedure.

### Cell cycle analysis

Cell cycle progression was analysed under different conditions through staining with propidium iodide (PI) by means of flow cytometry. 1 × 10^6^ cells from each sample were collected in ice-cold PBS and were then fixed in 70% cold ethanol for 1 h at 4 °C. Following further washes, DNA was stained by addition of PI solution (0.1% (v/v) Triton X-100, 10 μg/ml PI, and 100 μg/ml DNase-free RNase A in PBS) for 20 min at RT. Propidium iodide intensity was measured by FACS flow cytometer system (Becton Dickinson, CA, USA) and data were processed using Mod Fit v 4.0 program.

### RNA Isolation and qRT-PCR

Total RNA from three mentioned groups were isolated using TRIzol reagent (Ambion, Burlington, Canada) and revers transcribed by Takara cDNA synthesis kit (Takara, Otsu, Japan), based on manufacturer’s instruction.

Quantitative PCR was performed using SYBR Premix Ex Taq II (TaKaRa, Otsu, Japan) in a Rotor-Gene 6000 Real Time PCR System (Corbett, Sydney, Australia). The gene expression level was quantified via the ddCt method relative to the *GAPDH,* as the internal housekeeping gene. All gene-specific primers are listed in Supplementary Table S2.

### Western blot analysis

Activity of PI3K/AKT, MEK/ERK and JAK/STAT pathways were evaluated by comparing the expression of total and phosphorylated proteins using western blot analyses. HL-60 Cells were incubated with fenbendazole or DMSO for 5 days, along with untreated cells, as previously described. At the end of incubation time, whole-cell extracts were collected, using RIPA lysis buffer (Beyotime, Shanghai, China), according to the manufacturer’s instruction. The cells were washed twice with PBS, resuspended in ice-cold RIPA buffer (containing Tris HCL 7.6, NACL, NP-40, sodium deoxycholate, and SDS) enriched with a cocktail of protease (Melford, Ipswich, UK) and phosphatase inhibitors (Sigma, MO, USA) and incubated on ice for 10 min. Cell derbies were removed following centrifugation under 8000*g* for 10 min and suspended proteins were quantified via Bradford assay (Bio-Rad, WA, USA) according to the manufacturer’s instructions.

For western blotting, the separation of proteins was achieved in 15% SDS–polyacrylamide gel, which was followed by protein transfer onto a PVDF membrane. Subsequently, nonspecific binding sites were blocked with 5% BSA in TBS-tween buffer (10 mM Tris base, 150 mM NaCl, 0.05% [v/v] Tween 20) for overnight at 4^◦C^. Afterward, membranes were incubated with the appropriate primary antibodies diluted in blocking buffer for 2 h at room temperature. Next, the blots were washed three times in washing buffer (0.01%Tween20) and probed with HRP-conjugated secondary antibodies for 45 min at room temperature. The chemiluminescence detection of specific bands was performed using enhanced chemiluminescence (ECL) substrate (Amersham, NJ, USA) and densitometric analysis of the images were carried out image J analysis software (version 1.42). All primary and secondary antibodies are listed in Supplementary Table S3.

### Statistical analysis of cell viability curves

Data are expressed as mean ± SEM. Statistical analysis for the cell viability curves was done using GraphPad Prism 6.01 (GraphPad Software, San Diego, CA, USA).

### Ethics approval and consent to participate.

All experimental protocols were approved by Ethics committee of Royan Institute (IR.ACECR.ROYAN.REC.1397.142). All methods were carried out in accordance with relevant guidelines and regulations.

## Supplementary Information

Supplementary Information 1.

## Data Availability

The code for reproducing results is accessible here: https://github.com/yk313-ab454/LeukaemiaDifferentiationTherapy.

## References

[1] De Thé H (2018). Differentiation therapy revisited. Nat. Rev. Cancer.

[2] Nowak D, Stewart D, Koeffler HP (2009). Differentiation therapy of leukemia: 3 decades of development. Blood.

[3] Leszczyniecka M, Roberts T, Dent P, Grant S, Fisher PB (2001). Differentiation therapy of human cancer: Basic science and clinical applications. Pharmacol. Ther..

[4] Jarada TN, Rokne JG, Alhajj R (2020). A review of computational drug repositioning: Strategies, approaches, opportunities, challenges, and directions. J. Cheminform..

[5] Keenan AB (2019). Connectivity mapping: Methods and applications. Annu. Rev. Biomed. Data Sci..

[6] Kalantarmotamedi Y, Eastman RT, Guha R, Bender A (2018). A systematic and prospectively validated approach for identifying synergistic drug combinations against malaria. Malar. J..

[7] Musa A (2018). A review of connectivity map and computational approaches in pharmacogenomics. Brief. Bioinform..

[8] Lamb J (2006). The Connectivity Map: Using gene-expression signatures to connect small molecules, genes, and disease. Science.

[9] Iorio F (2010). Discovery of drug mode of action and drug repositioning from transcriptional responses. PNAS.

[10] Lamb J (2007). The connectivity map: A new tool for biomedical research. Nat. Rev. Cancer.

[11] Koutsoukas A (2013). In silico target predictions: Defining a benchmarking data set and comparison of performance of the multiclass Naïve Bayes and parzen-rosenblatt window. J. Chem. Inf. Model..

[12] Brum AM (2018). Using the connectivity map to discover compounds influencing human osteoblast differentiation. J. Cell. Physiol..

[13] Christodoulou E (2019). Identification of drugs for leukaemia differentiation therapy by network pharmacology. bioRxiv.

[14] Watson RWG (1997). Granulocytic differentiation of HL-60 cells results in spontaneous apoptosis mediated by increased caspase expression. FEBS Lett..

[15] Edgar R, Domrachev M, Lash AE (2002). Gene Expression Omnibus: NCBI gene expression and hybridization array data repository. Nucleic Acids Res..

[16] Subramanian A, Tamayo P, Mootha VK, Mukherjee S, Ebert BL (2005). Gene set enrichment analysis: A knowledge-based approach for interpreting genome-wide. PNAS.

[17] Davis AP (2013). The comparative toxicogenomics database: Update 2013. Nucleic Acids Res..

[18] Zhelev Z (2004). Phenothiazines suppress proliferation and induce apoptosis in cultured leukemic cells without any influence on the viability of normal lymphocytes, Phenothiazines and leukemia. Cancer Chemother. Pharmacol..

[19] Gaulton A (2012). ChEMBL: A large-scale bioactivity database for drug discovery. Nucleic Acids Res..

[20] Wang X, Wu Q, Zhang L, Wu Y, Shu Y (2010). Wortmannin induced apoptosis of leukemia cells by reducing PI3K/Akt. Chin. German J. Clin. Oncol..

[21] Li Y (2019). Mebendazole for differentiation therapy of acute myeloid leukemia identified by a lineage maturation index. Sci. Rep..

[22] Freisleben F (2019). Mebendazole mediates its anti-leukemic effects by proteasomal degradation of GLI transcription factors via inhibition of HSP70/90-chaperone activity in acute myeloid leukemia in a preclinical and clinical setting. Blood.

[23] Narasimhan K (2015). Genistein exerts anti-leukemic effects on genetically different acute myeloid leukemia cell lines by inhibiting protein synthesis and cell proliferation while inducing apoptosis—molecular insights from an iTRAQ quantitative proteomics study. Oncoscience.

[24] Takahashi T, Kobori M, Shinmoto H, Tsushida T (1998). Structure-activity relationships of flavonoids and the induction of granulocytic- or monocytic-differentiation in HL60 human myeloid leukemia cells. Biosci. Biotechnol. Biochem..

[25] Constantinou A, Kiguchi K, Huberman E (1990). Induction of differentiation and DNA strand breakage in human HL-60 and K-562 leukemia cells by genistein. CANCER Res..

[26] Laverdière I (2018). Leukemic stem cell signatures identify novel therapeutics targeting acute myeloid leukemia. Blood Cancer J..

[27] Collins SJ, Ruscetti FW, Gallagher RE, Gallo RC (1979). Normal functional characteristics of cultured human promyelocytic leukemia cells (HL-60) after induction of differentiation by dimethylsulfoxide. J. Exp. Med..

[28] Nath J (2020). Drug repurposing and relabeling for cancer therapy: Emerging benzimidazole antihelminthics with potent anticancer effects. Life Sci..

[29] Dogra N, Mukhopadhyay T (2012). Impairment of the ubiquitin-proteasome pathway by methyl N-(6-phenylsulfanyl-1H-benzimidazol-2-yl)carbamate leads to a potent cytotoxic effect in tumor cells: A novel antiproliferative agent with a potential therapeutic implication. J. Biol. Chem..

[30] Gao P, Dang CV, Watson J (2008). Unexpected antitumorigenic effect of fenbendazole when combined with supplementary vitamins. J. Am. Assoc. Lab. Anim. Sci..

[31] Duan Q, Liu Y, Rockwell S (2014). Fenbendazole as a potential anticancer drug. Anticancer Res..

[32] Hartsink-Segers SA (2013). Aurora kinases in childhood acute leukemia: The promise of aurora B as therapeutic target. Leukemia.

[33] Yu C (2004). Induction of apoptosis in human leukemia cells by the tyrosine kinase inhibitor adaphostin proceeds through a RAF-1/MEK/ERK- and AKT-dependent process. Oncogene.

[34] Zebisch A (2012). Frequent loss of RAF kinase inhibitor protein expression in acute myeloid leukemia. Leukemia.

[35] Qi J (2019). Selective inhibition of Aurora A and B kinases effectively induces cell cycle arrest in t(8;21) acute myeloid leukemia. Biomed. Pharmacother..

[36] Goldenson B, Kirsammer G, Stankiewicz MJ, Wen QJ, Crispino JD (2015). Aurora kinase a is required for hematopoiesis but is dispensable for murine megakaryocyte endomitosis and differentiation. Blood.

[37] Kim SJ (2012). Aurora A kinase expression is increased in leukemia stem cells, and a selective Aurora A kinase inhibitor enhances Ara-C-induced apoptosis in acute myeloid leukemia stem cells. Korean J. Hematol..

[38] Schwäble J (2005). RGS2 is an important target gene of Flt3-ITD mutations in AML and functions in myeloid differentiation and leukemic transformation. Blood.

[39] Gornati R (2007). Evaluation of SAT-1, SAT-2 and GalNAcT-1 mRNA in colon cancer by real-time PCR. Mol. Cell. Biochem..

[40] Huang Y (2006). Antileukemic roles of human phospholipid scramblase 1 gene, evidence from inducible PLSCR1-expressing leukemic cells. Oncogene.

[41] Kabir NN, Rönnstrand L, Kazi JU (2013). Deregulation of protein phosphatase expression in acute myeloid leukemia. Med. Oncol..

[42] Huang L (2012). Expression feature of CD3, FcεRIγ, and Zap-70 in patients with chronic lymphocytic leukemia. Hematology.

[43] McMahon M, Ayllón V, Panov KI, O’Connor R (2010). Ribosomal 18 S RNA processing by the IGF-I-responsive WDR3 protein is integrated with p53 function in cancer cell proliferation. J. Biol. Chem..

[44] Hölzel M (2005). Mammalian WDR12 is a novel member of the Pes1-Bop1 complex and is required for ribosome biogenesis and cell proliferation. J. Cell Biol..

[45] Bentley DL, Groudine M (1986). A block to elongation is largely responsible for decreased transcription of c-myc in differentiated HL60 cells. Nature.

[46] Monaco SE, Angelastro JM, Szabolcs M, Greene LA (2007). The transcription factor ATF5 is widely expressed in carcinomas, and interference with its function selectively kills neoplastic, but not nontransformed, breast cell lines. Int. J. Cancer.

[47] Park S (2010). Role of the PI3K/AKT and mTOR signaling pathways in acute myeloid leukemia. Haematologica.

[48] Coller HA (2000). Expression analysis with oligonucleotide microarrays reveals that MYC regulates genes involved in growth, cell cycle, signaling, and adhesion. Proc. Natl. Acad. Sci..

[49] Riccardi C, Nicoletti I (2006). Analysis of apoptosis by propidium iodide staining and flow cytometry. Nat. Protoc..

[50] Lin F (2019). STAT3-induced SMYD3 transcription enhances chronic lymphocytic leukemia cell growth in vitro and in vivo. Inflamm. Res..

[51] Shi Y (2018). Roles of STAT3 in leukemia (review). Int. J. Oncol..

[52] Yoyen-Ermis D (2019). Myeloid maturation potentiates STAT3-mediated atypical IFN-γ signaling and upregulation of PD-1 ligands in AML and MDS. Sci. Rep..

[53] Zhu Q (2014). The IL-6-STAT3 axis mediates a reciprocal crosstalk between cancer-derived mesenchymal stem cells and neutrophils to synergistically prompt gastric cancer progression. Cell Death Dis..

[54] Jacobson EC (2020). Hi-C detects novel structural variants in HL-60 and HL-60/S4 cell lines. Genomics.

[55] Dwivedi P, Greis KD (2017). Granulocyte colony-stimulating factor receptor signaling in severe congenital neutropenia, chronic neutrophilic leukemia, and related malignancies. Exp. Hematol..

[56] Miranda MB, McGuire TF, Johnson DE (2002). Importance of MEK-1/2 signaling in monocytic and granulocytic differentiation of myeloid cell lines. Leukemia.

[57] Miranda MB, Johnson DE (2007). Signal transduction pathways that contribute to myeloid differentiation. Leukemia.

[58] Dergachev V, Benhar I (2015). Resistance to Immunotoxins in Cancer Therapy.

[59] Martelli AM (2006). Phosphoinositide 3-kinase/Akt signaling pathway and its therapeutical implications for human acute myeloid leukemia. Leukemia.

[60] Zhu QS, Robinson LJ, Roginskaya V, Corey SJ (2004). G-CSF-induced tyrosine phosphorylation of Gab2 is Lyn kinase dependent and associated with enhanced Akt and differentiative, not proliferative, responses. Blood.

[61] Matkovic K, Brugnoli F, Bertagnolo V, Banfic H, Visnjic D (2006). The role of the nuclear Akt activation and Akt inhibitors in all-trans-retinoic acid-differentiated HL-60 cells. Leukemia.

[62] Lal L (2005). Activation of the p70 S6 kinase by all-trans-retinoic acid in acute promyelocytic leukemia cells. Blood.

[63] Touw IP, Palande K, Beekman R (2013). Granulocyte colony-stimulating factor receptor signaling. Implications for G-CSF responses and leukemic progression in severe congenital neutropenia. Hematol. Oncol. Clin. N. Am..

[64] Avalos B (1996). Molecular analysis of the granulocyte colony-stimulating factor receptor. Blood.

[65] Semerad CL, Liu F, Gregory AD, Stumpf K, Link DC (2002). G-CSF is an essential regulator of neutrophil trafficking from the bone marrow to the blood. Immunity.

[66] Dong F, Larner AC (2000). Activation of Akt kinase by granulocyte colony-stimulating factor (G-CSF): Evidence for the role of a tyrosine kinase activity distinct from the Janus kinases. Blood.

[67] Bender A, KalantarMotamedi Y, Peymani M, Nasr-Esfahani MH (2015). Computational methods for small molecule selection in stem cell differentiation. Eur. Pharm. Rev..

[68] Hochreiter S, Clevert D-A, Obermayer K (2006). A new summarization method for Affymetrix probe level data. Bioinformatics.

[69] Talloen W (2007). I/NI-calls for the exclusion of non-informative genes: A highly effective filtering tool for microarray data. Bioinformatics.

[70] Shannon P (2003). Cytoscape: A software environment for integrated models of biomolecular interaction networks. Genome Res..

[71] Calderone A, Castagnoli L, Cesareni G (2013). mentha: A resource for browsing integrated protein-interaction networks. Nat. Methods.

[72] Reich M (2006). GenePattern 2.0. Nat. Genet..

[73] Maere S, Heymans K, Kuiper M (2005). BiNGO: A Cytoscape plugin to assess overrepresentation of gene ontology categories in biological networks. Bioinformatics.

[74] Hosseini Farahabadi SS, Ghaedi K, Shoaraye-nejati A, Nasr-Esfahani MH (2020). Full small molecule conversion of human fibroblasts to neuroectodermal cells via a cocktail of Dorsomorphin and Trichostatin A. Regen. Ther..

[75] Olins AL, Buendia B, Herrmann H, Lichter P, Olins DE (1998). Retinoic acid induction of nuclear envelope-limited chromatin sheets in HL-60 ing separable mechanisms for ELCS formation and. Exp. Cell Res..

[76] Qi S-N, Song L-J, Chen Y, Jing Y-X (2010). Reversal of mdr1-mediated multidrug resistance in human leukemia cells by a new spin-labeled derivative of podophyllotoxin. Pharmazie.

[77] Ringshausen I, Oelsner M, Bogner C, Peschel C, Decker T (2008). The immunomodulatory drug Leflunomide inhibits cell cycle progression of B-CLL cells. Leukemia.

[78] Urbaniak A (2020). Carbamate derivatives of colchicine show potent activity towards primary acute lymphoblastic leukemia and primary breast cancer cells—in vitro and ex vivo study. J. Biochem. Mol. Toxicol..

[79] Popper LD, Batra SC (1993). Release of intracellular calcium by prenylamine in human ovarian tumour cells. Cancer Lett..

[80] Gomez GA, Sokal JE, Aungst CW (1978). Chemotherapy of the terminal phase of chronic myelocytic leukemia with combinations of colchicine derivatives and purine analogs. Leuk. Res..

[81] Barry MA, Reynolds JE, Eastman A (1993). Etoposide-induced apoptosis in human HL-60 cells is associated with intracellular acidification. Cancer Res..

[82] Tiwari S (2005). Type 4 cAMP phosphodiesterase (PDE4) inhibitors augment glucocorticoid-mediated apoptosis in B cell chronic lymphocytic leukemia (B-CLL) in the absence of exogenous adenylyl cyclase stimulation. Biochem. Pharmacol..

[83] Beswick RW, Ambrose HE, Wagner SD (2006). Nocodazole, a microtubule de-polymerising agent, induces apoptosis of chronic lymphocytic leukaemia cells associated with changes in Bcl-2 phosphorylation and expression. Leuk. Res..

[84] Warrell RP (1991). Differentiation therapy of acute promyelocytic leukemia with tretinoin (all-trans-retinoic acid). N. Engl. J. Med..

[85] Li W (2011). Genistein inhibited proliferation and induced apoptosis in acute lymphoblastic leukemia, lymphoma and multiple myeloma cells in vitro. Leuk. Lymphoma.

[86] Lukinović-Škudar V, Banfić H, Višnjić D (2011). Different effects of phosphatidylinositol 3-kinase inhibitor LY294002 and Akt inhibitor SH-5 on cell cycle progression in synchronized HL-60 leukemia cells. Period. Biol..

[87] Gout PW, Buckley AR, Simms CR, Bruchovsky N (2001). Sulfasalazine, a potent suppressor of lymphoma growth by inhibition of the x-c cystine transporter: A new action for an old drug. Leukemia.

[88] Ishihara K, Hong J, Zee O, Ohuchi K (2004). Possible mechanism of action of the histone deacetylase inhibitors for the induction of differentiation of HL-60 clone 15 cells into eosinophils. Br. J. Pharmacol..

[89] Inayat-Hussain SH, McGuinness SM, Johansson R, Lundstrom J, Ross D (2000). Caspase-dependent and -independent mechanisms in apoptosis induced by hydroquinone and catechol metabolites of remoxipride in HL-60 cells. Chem. Biol. Interact..

