# Supplementary material for: Transcriptional drug repositioning and cheminformatics approach for differentiation therapy of leukaemia cells
Source: Sci Rep. 2021 Jun 15;11:12537. doi: 10.1038/s41598-021-91629-x (PMC8206077; doi:10.1038/s41598-021-91629-x)
Supplement: Supplementary file 1 — Supplementary Information 1. [file 41598_2021_91629_MOESM1_ESM.pdf]

Transcriptional drug repositioning and cheminformatics approach for differentiation therapy of leukaemia cells

Yasaman KalantarMotamedi[1+], Fatemeh Ejeian[2+], Faezeh Sabouhi[3,2+], Leila Bahmani[2,4], Alireza Shoaraye Nejati[2], Aditya Mukund Bhagwat[5], Ali Mohammad Ahadi[3], Azita Parvaneh Tafreshi[4], Mohammad Hossein Nasr-Esfahani[2,*] & Andreas Bender[1,*]

[1]Centre for Molecular Informatics, Department of Chemistry, University of Cambridge, Lensfield Road, Cambridge CB2 1EW, United Kingdom.

[2]Department of Animal Biotechnology, Cell Science Research Centre, Royan Institute for Biotechnology, ACECR, Isfahan, Iran

[3]Department of Genetics, Faculty of Science, Shahrekord University, Shahrekord, Iran

[4] Molecular Medicine Department, Institute of Medical Biotechnology, National Institute of Genetic Engineering and Biotechnology, Tehran, Iran

[5]Open Analytics, Jupiterstraat 20, 2600 Antwerp, Belgium

[+]These authors contributed equally.

*Corresponding author's e-mail:

ab454@cam.ac.uk (AB),

mh.nasr-esfahani@royaninstitute.org (MN)

Total AKT (60 kDa).
Labels from tight are FDZ, DMSO, control.

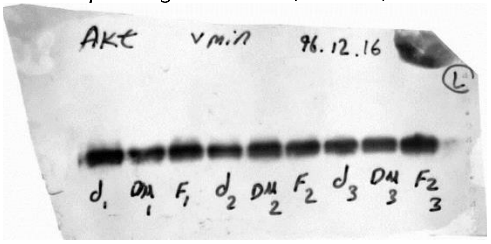

P-AKT (60 kDA)
Labels from right are control, DMSO, FDZ.

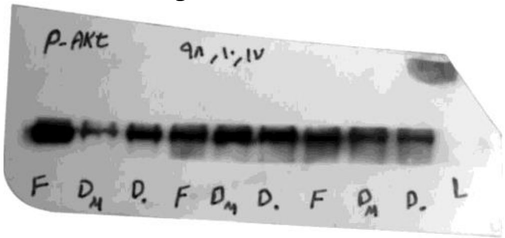

Total ERK (42 & 44 kDa)
Labels from right are control, DMSO, FDZ.

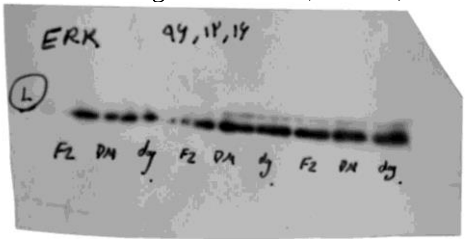

P-ERK (42 & 44 kDa)
Labels from left are control, DMSO, FDZ.

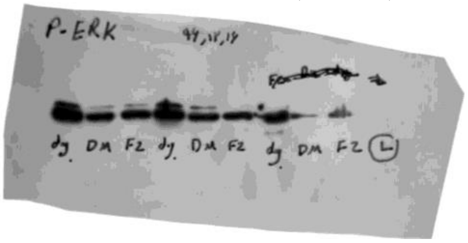

Total STAT3 (92 kDa)
Labels from left are control, DMSO, FDZ.

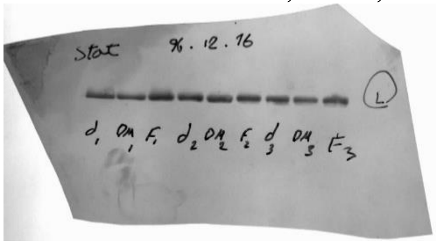

P-STAT3 (expected: 79 & 86 kDa)

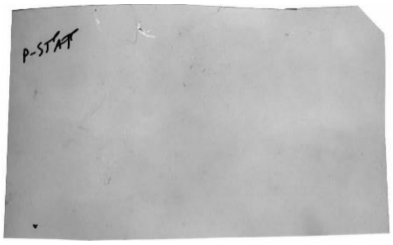

GAPDH (38 kDa)
Labels from left are control, DMSO, FDZ.

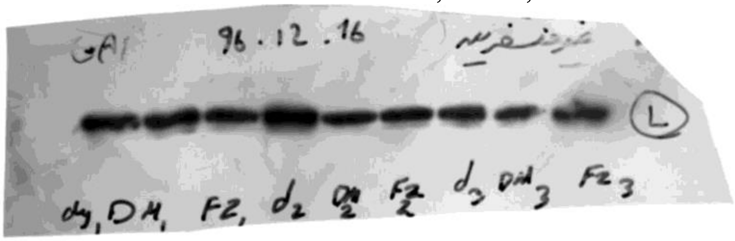

Supplementary Figure 1. Full-length blots for pathway analysis of HL-60 cells treated with DMSO and fenbendazole. For the western blot assay, untreated HL60 cells (Day 0) were considered as the negative control and the DMSO-treated cells were used as a positive control. Corresponding molecular weight for each specific antibody are labelled on full length blots based on the standard protein markers (pre-stained protein ladder, Ferments, 10 – 170 KDa). Quantitative comparisons were routinely carried out for three independent experimental groups evaluated on a single blot.

Supplementary Table 1: up/down gene signatures of fenbendazole (instance ID 2360) and leukaemia differentiation (HL60 vs. granulocytes).

FDZ up	FTH1, C3AR1, TIMP1, PTPRE, IER3, RGS2, PLSCR1, IL8, SAT1, IL10RA, ANXA1, FPR1, CCND3, FCER1G, CYTIP, SAMSN1, CD55, HBEGF, GPR183, LPXN
FDZ dow	TUBB, TUBB4B, TTC27, ORM1, NOP16, ADRB2, TUBB6, ATF5, ODC1, SLC25A32, MRTO4, WDR3, TIMM23, HK2, CTH, FAM46A, WDR12, MYC, COQ3, TUBA1A
Leukaemia up	RNU5D, KIAA0101, ASPM, MAD2L1, CENPF, POLQ, NME1, CDK1, HMMR, PSAT1, TOP2A, BUB1, RAD51AP1, DLGAP5, KIF11, WDHD1, DTL, HELLS, CDCA7, ARHGAP11A, TYMS, NCAPG, CCNB1, OVOS, C18ORF55, MCM6, PRG2, TAF4B, TTK, HIST1H3B, CDCA2, SCD, NUF2, KIF14, SPC25, TRIP13, ALDH18A1, HIST1H1B, KIF15, SNRPD1, KIF20B, GPR85, RPF2, ATIC, CEP55, RAD51, PLK4, SHCBP1, LRPPRC, CCDC26, PBK, CENPW, GGH, ARHGAP11B, BUB1B, CHEK1, CCNB2, GINS1, C2ORF43, XRCC2, SUCNR1, CENPE, SLC16A1, TPX2, BEX1, HSPA4L, C5ORF28, SLC27A2, POLR1E, MTX2, NOC3L, CCDC99, MPO, KIAA0020, CENPI, CDC45, KIF4A, DPH5, WDR12, CTPS, CNTNAP4, MND1, C12ORF48, PRC1, ALDH1L2, CKAP2L, HIST1H2AB, ARMC1, NAT10, ORC6, CDC6, KIF2C, KIF23, MRPL1, C20ORF103, ESCO2, PPAT, EXO1, RRM2, MRPS28, ZNHIT6, SNRPF, LPL, MELK, SLC35F2, MKI67, IPO5, LMAN1, FH, KCTD3, GXYLT2, ANLN, ORC1, ACACA, C4ORF43, FIGNL1, DKFZP686O24166, DSCC1, MPHOSPH6, LRRC34, RANBP1, PRAME, UMPS, KDELC1, UTP20, NUBPL, ME1, ELOVL6, TYW3, SMC2, IGSF10, TIMELESS, SPAG5, MYC, FASTKD2, GPNMB, AGPAT5, KIF18A, FBXO5, CCNE1, STRBP, CLSPN, NXF3, ABCE1, EXTL2, MNS1, MRPS33, WDR3, BLMH, BRIP1, PRDX4, SSRP1, SEH1L, SPATA5, KIF20A, FASTKD1, PAICS, LOC400986, IARS, MSH2, C21ORF45, MARS2, CA8, TRUB1, SAAL1, C16ORF88, C17ORF75, ESF1, STMN1, RPS29, PAK1IP1, GNL3, C3ORF26, SPC24, ZNF175, PSMD14, OIP5, CTSG, USP14, POLR1C, ZNF566, GTF2H3, MRPS23, CDC25A, SACS, OXCT1, TRMT5, BLM, CHEK2, C15ORF42, CHD1L, DIAPH3, ENOSF1, CIT, EPRS, DTNA, METAP2, C1ORF163, POLA1, UBE2T, TRIT1, ASS1, RMND1, RPL7L1, PFDN4, TMEM97, NCAPG2, MMS22L, ZNF749, CTH, VIT, SCML2, MRPL3, CLDN12, SLC7A1, LSM5, DNAJC12, UBA5, POLR1B, TRAP1, NDFIP2, CCNA2, DHCR24, CENPL, MCM4, PUS7, NDUFAF4, XPO4, TARS, FANCI, HIST1H4L, MCM3, NUDCD1, C12ORF45, DDX10, THG1L, NAA25, GSTCD, POLR1A, ZNF670, GAS2L3, PTPLAD1, MTBP, MAK16, C13ORF38, HIST1H2AH, MTHFD1, DCLRE1A, EPB41L3, GFM1, CDKN3, BCKDHB, EXOSC8, IGF2BP1, ZNF519, SLC1A4, N6AMT2, LRRCC1, SQLE, PHGDH, MRPL24, KCNQ5, ERI2, MPP6, AARS, SUV39H2, C12ORF11, NIPSNAP3B, PARP2, C13ORF34, KDM1A, MRPL35, URB2, TIMM13, FABP5, DLEU1, C4ORF21, THUMPD2, COG2, RUVBL1, CDC7, TSR1, MRPS35, SERPINB10, LARS, SMARCAD1, TOMM70A, MTERFD1, SHMT2, ZWINT, PAR5, RTN4IP1, ZNF221, AURKA, PAAF1, EDIL3, FOXM1, TARBP1, NOP16, MRPL15, COX6C, ETV5, CHML, URB1, TMEM67, QDPR, BRCA2, MRRF, FADS1, IMMP2L, POGLUT1, NAA15, USP13, UGGT2, RPL22, GART, LTV1, CCNB1IP1, NOB1, DHX32, CASC5, PTGR1, GLMN, MRTO4, RRM1, BMS1P1, ATAD5, INTS2, KIAA0090, HEATR1, ZNF215, METTL2A, APOA1BP, NUSAP1, C1ORF135, NCAPH, ATPBD4, CLEC5A, MCM10, HSPH1, KIAA1586, PHF14, KCNK5, FANCC, NETO2, FAM3C, BDH1, SRPRB, NUP205, AKAP1, SATB2, GINS4, CENPH, FAM72D, C5ORF13, OCRL, C3ORF78, GNPNAT1, FBXO4, MAGOHB, GTF3C3, BZW2, PDIA5, BRIX1, SNORD101, AFG3L2, IMMP1L, WDR17, UCK2, MDN1, DNAJC11, C10ORF2, TMEM38B, UNG, FAT1, MDH1, FLT3, GINS3, WDR76, STIL, UBE2E2, CEP290, CCL2, KDELC2, E2F8, PALB2, TRMT1, PRIM1, GPT2, WDR43, ZNF229, BCS1L, NUDT6, CENPJ, SGOL2, LDHB, C5ORF25, ASCC3, ST7, RTTN

Leukaemia down	PIP5K1B, MIR21, MIR23A, CCIN, C12ORF35, CD3E, CYP4F3, BAIAP3, INADL, FAM13A, HPSE, MIR103-2, MAP1LC3B, TXNDC3, RIMKLB, TANK, MIR29C, NAIP, Mar-01, SOCS3, CSF1, MIRLET7G, TRIM6-TRIM34, CARD17, TCN1, CAST, DLEC1, PRF1, IRF2, FAM151B, DYNLT1, CD177, NHSL2, HLA-H, ZDHHC18, ZNF429, OR52K2, CAPRIN2, IL32, MIR223, SLC43A2, ALS2CR12, C2ORF61, PRDM1, SNORA16B, SEC14L1, C14ORF148, CPNE8, SELP, UBE2D1, MMP9, MYBL1, KRTAP10-5, PTPLAD2, ZBP1, TNIK, RTDR1, SPRY3, FAS, OR52K1, FAM63A, MANSC1, ACPL2, FAM174A, ITK, ZNF44, MICALCL, BTN3A1, NR4A2, RNU4-2, PFKFB4, BTG2, SLED1, STX11, MGC39372, PELI1, TREML3, IFITM2, VCAN, OR2T3, AGPAT9, IGSF6, HLA-DPA1, ADAMTSL4, CYTIP, Mar-08, C1ORF183, CD3G, MIR30E, HLA-DQB1, IL1RN, SPOCK2, SIGLEC10, QPCT, KRCC1, LOC100131541, TGFA, GIMAP7, S100A12, MMP25, LPAR1, FAM71F2, DPEP2, TNFSF8, CARD16, MEFV, BEST1, STAT4, IL1B, PARP9, IFIH1, CXORF65, MIR101-1, RBP7, ZNF117, NOV, APC, IDO1, OSM, G0S2, ZNF626, PACS1, OCR1, CASS4, SERPINB9, MAPRE3, ITGAX, WWC3, XKR8, GRK5, CPNE3, SERPING1, SEC61A2, PTTG2, KIF13A, TMCC1, ARRDC3, FAIM3, OASL, FAM106A, YPEL3, CYP4F12, PYGM, LYVE1, LOC284751, CCDC146, PILRA, TLR6, TMEM71, FLJ27255, FXR2, SP110, HLA-DQA1, MX1, VSTM1, IL18R1, DDX58, MX2, OAS3, RASGRP1, TMEM88, CMTM2, LRRC6, ICAM1, LILRA1, CXCL1, SLFN5, LOC162632, FYB, HBA1, GAB1, NFKBIZ, SAMD9L, NLRP6, EXT1, RORA, C12ORF55, SPATA13, HCP5, ECE1, SLAIN1, HIST1H1T, TIAM2, DHRS9, C7ORF53, C17ORF91, MIR147, KIAA0319, ZNF486, ARG1, RASGRP4, DYSF, DSC2, LIPN, CNTNAP3, LRRC4, DAPK1, ATHL1, GPR65, ZNF185, FPR2, GBP2, SLC44A2, NCOA1, TMEM45B, RARRES3, TLR8, GPR97, BCL6, GCH1, TMCC3, IFIT5, GDPD3, LOC100127886, KIAA0513, OAS2, CAMK1D, SLC35D2, LILRB2, CLEC4A, TNFRSF9, PRR5L, HLA-F, SYNE1, FCHO2, ISG20, C5AR1, GBP1, TNFSF10, CYP27A1, SLAMF7, LRRN1, ZBTB38, MAML2, TRAT1, GZMK, NLRP1, SYTL3, CXCR2, BMPR2, BMX, RGS2, PRKCH, TLR1, EFHC2, PARP12, MIR29A, LOC93432, FCGR2A, PXN, IL1R1, CD96, FAM49A, NOD2, REM2, IFIT3, APOBEC3G, NCRNA00282, GNG2, PI3, ZFYVE16, GPR109A, PDK4, F5, SIPA1L1, KLRK1, CLEC7A, TLR5, SPATA6, TP53INP1, BTNL8, GCM1, MIR29B2, TREM1, PLXDC2, TMEM140, AIM2, GLT1D1, PPBP, BCL2A1, LOC100131131, CLEC1A, FAM19A2, SLC11A1, CTNNA1, SLC46A3, REPS2, ATP8A1, ZFP36L1, JAK3, S100Z, GVINP1, HIST2H2BA, CYSLTR2, ZNF844, C1ORF26, F2RL1, C13ORF18, EPSTI1, HSPC159, LILRA3, MPP7, EMR3, C9ORF72, WLS, KCNJ2, CLEC2B, IL7R, IL1R2, CRISPLD2, IQSEC1, CD274, DUSP1, CD93, TC2N, CDKL5, TRPM6, NSUN7, CLEC4E, PTGS2, IL18RAP, RNF144B, TCP11L2, P2RY13, TMEM2, MPZL2, GPR155, PHOSPHO1, SECTM1, ADAM19, TRANK1, C6ORF204, CEACAM1, IFI44L, LILRB3, HIVEP2, TNFRSF10C, NUAK2, RSAD2, FFAR2, OMG, LOC400499, LRRK2, ALPL, NCRNA00189, CRISP3, IFI16, P2RY10, MAN1A1, SLC37A3, GPR109B, IFITM3, MEF2C, CCR3, AQP9, FAM65B, CLEC9A, C5ORF36, APOBEC3A, GPR77, GBP4, MAK, IFIT1, KRT23, P2RY14, VNN1, SNORD56B, FCGR3A, MBOAT2, KCNJ15, HORMAD1, CXCL16, CHST15, SLC40A1, VNN2, IFI44, JHDM1D, KIAA1324, SYNE2, CASP5, CREB5, TLR10, GRAMD1C, GIMAP4, HSPA1A, HBB, VNN3, LY96, GBP5, IFITM1, IFIT2, MME, LILRA5, MIR24-2, KIAA0040, STEAP4, SULT1B1, PLBD1, SELL, CXCR1

Supplementary table 2. Primer sequence details for analysed genes

Gene	Primer sequence (5´-3´)	Product length (bp)	Accession number
AKT1	F 5`-GGCGAGCTGTTCTTCCACCTGTCC-3`<br>R 5`-TCTGTGATCTTAATGTGCCCGTCC-3`	179	NM_001014431.2
STAT3	F 5`-5`-CACCTTCCTGCTAAGATTCA-3`<br>R 5`-TTACCGCTGATGTCCTTCTC-3`	81	NM_001369512.1
ERK	F 5`-TGGTGTGCTCTGCTTATG-3`<br>R 5`-AGTAGGTCTGGTGCTCAA-3`	81	NM_001038663.1
GCSF	F 5`-AAGACAGGGAAGAGCAGAAC-3`<br>R 5`-TACAGGCAGGAGAATGAAAC-3`	78	NM_000759.4
GCSFR	F 5`-GGAGGATGGAACAGAATGGGAG-3`<br>R 5`-GGTACAAGGGAGTCACGATGAT-3`	96	NM_000760.4
C-MYC	F 5`-AGCATACATCCTGTCCGTCCA-3`<br>R 5`-TTACGCACAAGAGTTCCGTAGCTG-3`	121	NM_002467.4
CD55	F 5`-AAGGCTAAATTCTGCATCCCT-3`<br>R 5`-TTCTCTTCTGTAACCTGGACGG-3`	103	NM_000574.5
ATF5	F 5`-TCAATGTCTATGCCCGTCACA-3`<br>R 5`-CTCTATCCTGTCGCCACTCC-3`	146	NM_001193646.1
WDR12	F 5`-TCTTTGTATTTCCGCCTCTC-3`<br>R 5`-TGCTTGACTAACGCCTTG-3`	134	NM_018256.3
FPR1	F 5`-ATTGCCAGTTATCATTCGTGT-3`<br>R 5`-TATCCTCTCTTTAGGGTCGTT-3`	103	NM_001193306.2
RGS2	F 5`-TGTTTACTATGTGCAACGGTA-3`<br>R 5`-GGCACTCATAACGGACAC-3`	168	NM_002923.3
FTH1	F 5`-TGAATGAGCAGGTGAAAGC-3`<br>R 5`-GTCAAAGAGATATTCCGCCAAG-3`	101	NM_002032.2
PLSCR1	F 5`-CCAGTTCCCTTTAGACCTTGA-3`<br>R 5`-TAATCCACTACCACACTCCT-3`	128	NM_021105.2
MRTO4	F 5`-CCAAATCCAAGCGCGACA-3`<br>R 5`-GTCCACACATTTCCGAAGCTC-3`	101	NM_016183.3
TUBB	F 5`-CCACGTCTCCATTTCTTTATGCCT-3`<br>R 5`-ACCTCCTTCATGGACATCCGAC-3`	174	NM_001293212.2

Supplementary table 3. General characters of specific primary and secondary antibodies

Antibody	Cat-number	Company	Dilution
Mouse anti GAPDH antibody	SC-47724	Santa Cruz Biotech	1:5000
Rabbit anti-AKT antibody	SC-8312	Santa Cruz Biotech	1:100
Rabbit anti-p-AKT antibody	SC-514032	Santa Cruz Biotech	1:200
Mouse Anti-Stat3 antibody	BD610189	biosciences	1:200
rabbit anti p-stat3	#9145	Cell signaling	1:100
Rabbit anti-ERK antibody	#4695	Cell Signaling	1:200
Rabbit anti-p-ERK antibody	#9101	Cell Signaling	1:200
HRP-conjugated goat anti-rabbit IgG	SC2301	Santa Cruz	1:16000
HRP-conjugated goat anti-mouse IgG	P0447	Dako	1:5000
